# ROS Overproduction Sensitises Myeloma Cells to Bortezomib-Induced Apoptosis and Alleviates Tumour Microenvironment-Mediated Cell Resistance

**DOI:** 10.3390/cells9112357

**Published:** 2020-10-26

**Authors:** Mélody Caillot, Florence Zylbersztejn, Elsa Maitre, Jérôme Bourgeais, Olivier Hérault, Brigitte Sola

**Affiliations:** 1 INSERM, UNICAEN, Normandie Université, F-14000 Caen, France; melody.caillot@unicaen.fr (M.C.); 21900284@etu.unicaen.fr (F.Z.); maitre-e@chu-caen.fr (E.M.); 2 Laboratoire d’Hématologie, CHU Côte de Nacre, F-14000 Caen, France; 3 Service d’Hématologie Biologique, CHRU de Tours, F-37000 Tours, France; j.bourgeais@chu-tours.fr (J.B.); or olivier.herault@univ-tours.fr (O.H.); 4 LNox, CNRS, Université de Tours, F-37000 Tours, France

**Keywords:** reactive oxygen species, resistance, bortezomib, auranofin, antioxidant enzymes, survival, 3-D culture

## Abstract

Multiple myeloma (MM) is a plasma cell neoplasm that remains incurable due to innate or acquired resistance. Although MM cells produce high intracellular levels of reactive oxygen species (ROS), we hypothesised that they could remain sensitive to ROS unbalance. We tested if the inhibition of ROS, on one hand, or the overproduction of ROS, on the other, could (re)sensitise cells to bortezomib (BTZ). Two drugs were used in a panel of MM cell lines with various responses to BTZ: VAS3947 (VAS), an inhibitor of NADPH oxidase and auranofin (AUR), an inhibitor of thioredoxin reductase (TXNRD1), an antioxidant enzyme overexpressed in MM cells. We used several culture models: in suspension, on a fibronectin layer, in coculture with HS-5 mesenchymal cells, and/or in 3-D culture (or spheroids) to study the response of MM primary cells and cell lines. Several MM cell lines were sensitive to VAS but the combination with BTZ showed antagonistic or additive effects at best. By contrast, in all culture systems studied, the combined AUR/BTZ treatment showed synergistic effects on cell lines, including those less sensitive to BTZ and primary cells. MM cell death is due to the activation of apoptosis and autophagy. Modulating the redox balance of MM cells could be an effective therapy for refractory or relapse post-BTZ patients.

## 1. Introduction

Multiple myeloma (MM) is a haematological malignancy characterised by the proliferation and the accumulation of clonal plasma cells in the bone marrow. Tumour cells overproduce a monoclonal immunoglobulin or a light chain that, ultimately cause immunodeficiency, renal failure, recurrent infections, and bone lesions [1]. The introduction of proteasome inhibitors (PIs), including bortezomib (BTZ) and carfilzomib (CFZ), has led to a significant benefit for the treatment of MM patients [2].

The 26S proteasome is the main protein degradation machinery in cell [3]. It is composed of the 20S catalytic core that directs peptide bond cleavage and the 19S complex that recognises ubiquitinylated substrates. PIs target, either irreversibly (BTZ) or reversibly (CFZ, ixazomib), the β5 catalytic subunit of the 20S core complex. Due to their rapid and elevated production of immunoglobulins, MM cells are hypersensitive to proteasome inhibition [4]. By inhibiting the proteasome machinery and preventing the degradation of misfolded proteins, PIs induce an endoplasmic reticulum (ER) stress and an unfolded protein response (UPR), leading tumour cells to apoptosis. Unfortunately, most MM patients relapse due to innate or acquired resistance [5]. Several mechanisms sustain BTZ/CFZ resistance such as: mutation within the *PSMB5* gene encoding the β5 subunit [6], paradoxical knockdown of 19S regulatory components [7,8], activation of the aggresome-autophagy pathway [9], up-regulation of heat-shock proteins and ER stress sensors [10], overexpression of the multi-drug transporter ABCB1 [11]. Furthermore, the interactions between MM cells and their microenvironment participate in PIs resistance through soluble factors (interleukin (IL)-6, vascular endothelial growth factor) and exosomes, adhesion proteins of the integrins family, and/or specific miRNAs [5]. A comparative proteomic profiling of refractory/relapsed MM patients has confirmed four types of biomarkers for BTZ resistance. They belong to proteins involved in: (a) proteasome function, (b) response towards oxidative stress, (c) defence response, and (d) apoptotic process [12].

All MM cells express one of the three cyclin D proteins and almost 50% of them express cyclin D1. Besides the regulation of cell cycle and cell proliferation, we previously reported that the overexpression of cyclin D1 unbalances the MM redox status by producing reactive oxygen species (ROS) in a NADPH oxidase (NOX)-dependent manner [13]. Moreover, cyclin D1 sensitises cells to CFZ by activating the UPR pathway [14]. The targeting of tumour cells by a ROS-mediated mechanism has been described as a fruitful approach [15]. We report here that VAS3947 (VAS), a pan-inhibitor of NOX, as single-agent, is potent to induce MM cells death but fails to synergise with BTZ. In contrast, auranofin (AUR), an inhibitor of the antioxidant thioredoxin reductase shows a strong anti-MM activity, synergises with BTZ, and alleviates BTZ intrinsec insensitivity as well as cell tumour microenvironment (TME)-mediated cell resistance. The efficacy of drugs combination was confirmed in primary samples from MM patients cultured in suspension, in coculture, or in 3-D. The question of whether to antagonise or otherwise promote an oxidative stress in a therapeutic anti-MM strategy is, at least, partially solved by our data.

## 2. Materials and Methods

### 2.1. Drugs

VAS3947 (VAS), a selective inhibitor of NOX, was purchased from Calbiochem (#532336, San Diego, CA, USA); bortezomib (or PS-341) was purchased from SelleckChem (S1013, Houston, TX, USA). *N*-acetyl-l-cysteine (NAC), an antioxidant, bafilomycin A1 (BafA1), an inhibitor of maturation of autophagic vacuoles, and auranofin (AUR), a selective thioredoxin reductase inhibitor, were purchased from Sigma-Aldrich (A7250, B1793, and A6733, respectively, Saint Louis, MO, USA). Stock solutions (10 mM) were made using dimethylsulfoxide (DMSO) or ethanol (EtOH) as solvents. In turn, depending on the drugs, 0.01% DMSO or EtOH were used as vehicles.

### 2.2. Cell Lines and Culture Models

Nine MM cell lines belonging to various molecular subgroups were used in this study (Table S1). Cell line authentication was done by short tandem repeat (STR) profiling (DSMZ, Leibniz Institute). Cells were maintained in culture in RPMI 1640 medium supplemented with 10% foetal calf serum (FCS), 2 mM l-glutamine, and antibiotics (Lonza, Basel, Switzerland). Each batch of cells was maintained less than 2–3 months in culture. Mycoplasma infection was tested regularly by PCR.

Ninety-six-well plates were coated overnight at room temperature with fibronectin (FBN, 10 µg/mL in PBS, 100 µL/well, Sigma-Aldrich), then extensively washed before MM cells seeding. The human stromal cell line HS-5, obtained from the ATCC (CRL-11882), was maintained in Dulbecco’s modified Eagle’s medium containing antibiotics, l-glutamine, and 10% FCS (Lonza). For coculture experiments, HS-5 cells (9 × 10^4^ cells) were seeded in each well of 24-well plates and cultured for four days. MM cells (10^5^ cells/well) were seeded at that time directly on the monolayer for drug treatments. Plates were incubated for 1 h at 37 °C to allow MM cells’ attachment to the layer and cells were treated thereafter.

The method for 3-D culture was adapted from [16]. Briefly, 24-well plates were coated with 200 μL of reconstituted endosteum obtained by a mixture of FBN (77 μg/mL) and collagen I (29 μg/mL). MM cells (10^6^ cells in a volume of 40 μL of PBS) were suspended in a mixture of Matrigel (Corning), 1 mg/mL FBN, and 2 mg/mL collagen IV with a 4/2.5/1 ratio. Two hundred μL of this matrix were added to each well and incubated at 37 °C for 1 h before addition of complete medium. Drugs were added directly into the wells. For the dissociation of spheroids that formed in this 3-D culture, cells were incubated with a solution containing 5 mM EDTA, 1 mM sodium vanadate, and 1.5 mM sodium fluoride, then analysed.

### 2.3. Primary Samples, Treatments, and CD138 Expression Analyses

Primary neoplastic plasma cells were obtained at diagnosis from four patients of *Centre Hospitalo-Universitaire* of Caen. MM diagnosis was made in accordance with the International Myeloma Working Group criteria [17]. Informed consent was obtained from each patient in accordance with the guidelines of the local ethics policy and the Declaration of Helsinki. The clinical characteristics of MM patients are listed in Table S2. Mononuclear cells from bone marrow samples were isolated by Ficoll and directly cultured for 24 h in RPMI 1640 medium containing 10% FCS and 3 ng/mL recombinant IL6 (R&D Systems). Cells were then treated with AUR (0.25–5 μM) alone or in combination with BTZ (2.5–5 nM). Due to the limited number of tumour cells, experiments were not always performed in triplicate; the number of samples is clarified in the figure legend. After treatments, tumour cells were co-stained using a V450-conjugated anti-CD38 antibody (Ab, Clone HB7, #646852, BD Horizon) and a phycoerythrin(PE)-conjugated anti-CD138 Ab (A54190, IOTest, Beckman Coulter, Brea, CA, USA). CD38-positive cells were selected and cell death was measured by the loss of CD138 staining as described previously [18]. In two cases, we had enough CD38/CD138-positive primary cells to culture them in coculture with the HS-5 feeder layer as described previously (Pts # 3 and 4); in another one, we could maintain primary cells in spheroids (Pt # 1). In that case, purified mononuclear cells were cultured for six days then treated with drugs for 24 h. In these culture conditions, cell death was measured by the loss of CD138 staining after spheroid dissociation.

### 2.4. Cell Viability Assay

MM cell lines (5 × 10^4^ cells per condition) were seeded in 96-well plates and incubated for 48 h with vehicle, as a control, or drugs at the indicated doses. Cell viability was quantified with the CellTiter96^®^ Aqueous One Solution (MTS assay, Promega, Madison, WI, USA), according to the manufacturer’s instructions. The IC_50_ (index of cytotoxicity) that is the drug concentration that kills 50% of the cells after a 48 h-treatment, was calculated with the Prism software (v8.0, GraphPad, San Diego, CA, USA) and verified with the CompuSyn software (http://www.combosyn.org). This software was also used to calculate the Chou-Talalay combination index (CI) [19].

### 2.5. Assessment of Apoptosis

MM cells were treated with drugs (or vehicle) as indicated, then stained with 5 μg/mL Hoechst 33,342 (910–3015, ChemoMetec, Allerod, Denmark), 5 μg/mL propidium iodide (PI, 910-3016, ChemoMetec) and fluorescein isothiocyanate (FITC)-conjugated Annexin V (IM3546, Beckman Coulter), then analysed using the NucleoCounter NC-3000 image cytometer (ChemoMetec). Hoechst 33,342 stains the total cell population, annexin V stains both apoptotic and necrotic cells. Early apoptotic cells exclude PI, while late apoptotic cells stain positively for both annexin V and PI. The pan-caspase inhibitor Q-VD-OPh [quinoyl-valyl-*O*-methylaspartyl-(2,6-difluorophenoxy)-methyl ketone] was purchased from Sigma-Aldrich (SML0063). Cells were treated with 10 μM Q-VD-OPh for 1 h before the treatment with apoptosis inducers. At least 10^4^ cells were analysed for each set of culture condition. The experiment was carried out three times for LP1 cells and four times for KMS-12-PE with triplicate samples.

For each culture condition, MM cells were fixed in EtOH, then stained with DAPI (4′,6-diamidino-2-phenylindole) (910-3012, ChemoMetec), analysed for cell cycle distribution with the NucleoCounter NC-3000 using a pre-loaded protocol (two-step cell cycle analysis). Data were processed with the NucleoView software (ChemoMetec), exported, and analysed with the Kaluza software (Beckman Coulter). Cells with a sub-G1 DNA content were considered as apoptotic. At least 10^4^ cells were analysed for each set of culture condition and the experiment was carried out three times for each cell line with triplicate samples.

MM cells were treated with drugs or vehicle as before, stained with a PE-conjugated anti-APO2.7 Ab (IM2088U, IOTest, Beckman Coulter), and analysed by flow cytometry. For coculture experiments, HS-5 and MM cells were stained with APO2.7-PE and CD10-allophycocyanin (APC)-conjugated (B49223, IOTest, Beckman Coulter) Abs. Only CD10-negative cells corresponding to MM cells were analysed with the CytoFlex cytometer and the CytExpert software (Beckman Coulter). At least 10^4^ cells were analysed for each culture condition, the experiment has been done three times for each cell line with triplicate samples.

### 2.6. ROS Production Measurement

Intracellular ROS levels were measured with the oxidation-sensitive fluorescent CellROX™ Deep Red reagent (C10422, Invitrogen, Carlsbad, CA, USA) according to the manufacturer’s instructions. Treated cells were incubated with 5 μM reagent for 30 min at 37 °C and then analysed with the NucleoCounter NC-3000. We used a home-made protocol created on the FlexiCyte framework (ChemoMetec). Data were processed with the NucleoView software and analysed with the Kaluza software. The experiment was performed three times for all cell lines, except for LP1, only twice.

### 2.7. Immunoblotting

Cells were lysed with a lysis buffer containing 1% NP40, 10% glycerol, 0.05 M Tris pH7.5, 0.15 M NaCl, and a cocktail of protease and phosphatase inhibitors. Insoluble material was discarded and soluble proteins were recovered and quantified. The methods used for immunoblotting (IB) have been described in details elsewhere [20].

Activated (cleaved) caspase 3 is a hallmark of apoptosis induction. The cleavage of caspase 3 was analysed with anti-cleaved caspase 3 Abs (ab214430 from abcam, Cambridge, UK; or #9664 from Cell Signaling Technology, Danvers, MA, USA). To test for the induction of autophagy, we analysed the conversion from LC3B-I to LC3B-II with a specific Ab (ab51520, abcam). An anti-β-actin Ab (#4970, Cell Signaling Tech.) was used as a control of protein loading and transfer. The level of relevant proteins was estimated by densitometry (ChemiDoc XRS+, ImageLab software, Bio-Rad, Hercules, CA, USA) and normalised to the level of β-actin on three independent blots.

### 2.8. Semi-Quantitative RT-PCR Analyses

Semi-quantitative RT-PCR analyses was performed as previously described in Bustany et al. [13]. Total RNA was purified from cultured MM cell lines with the Trizol reagent (Invitrogen) according to the manufacturer’s instructions. The RNA was reverse-transcribed using the SuperScript^®^ VILO cDNA Synthesis Kit (Invitrogen). PCR primers and Universal Probe Libriray (UPL) probes were designed using ProbeFinder software (v1.5.1, Roche Applied Software, Penzberg, Germany) (Table S3). cDNAs, primers, probe, and LightCycler^®^ TaqMan^®^ Master mix were mixed in a final volume of 10 μL and PCR-amplified in a LightCycler^®^ 480 Instrument II (Roche) according to the manufacturer’s instructions. The Ct means of human *GAPDH*, *ACTB*, and *RPL13A* genes were used as endogenous control to normalise the expression of target genes. Each reaction condition was performed in triplicate. Relative gene expression was evaluated by the ΔCt method.

### 2.9. Statistical Analyses

The Student’s *t*-test was used to determine the significance of differences between two experimental groups. In some experiments comparing more than two groups, a one-way ANOVA test was performed. Data were analysed with the Prism software, with *p* < 0.05 (*) considered to be significant.

The Mann-Whitney test was used to determine the significance of GEP in MM patients according to their molecular sub-groups. Data were analysed with the Prism software, with *p* < 0.05 (*) considered to be significant.

## 3. Results

### 3.1. NOX Subunits Are Expressed in MM Patients and Cell Lines

We have previously reported that, in cyclin D1-expressing cells, ROS are produced through the activation of NOX. In turn, cyclin D1-expressing LP1 MM cells synthesized a high level of ROS, even in the absence of any stress [13]. In agreement with these data, we found that in cyclin D1-expressing LP1 cells, NOX2 (CYBB or gp91^phox^) and RAC were overexpressed (Figure S1a). Moreover, MM cell lines constitutively synthesise ROS and exhibit higher intrinsic oxidative stress than normal cells as do most cancer cells [13]. We confirmed that ROS was actively produced in MM cell lines by treating them with NAC and measuring intracellular ROS levels using an oxidation-sensitive fluorescent probe. The NAC-treatment decreased ROS level in all MM cell lines (Figure S1b).

**Figure 1:**
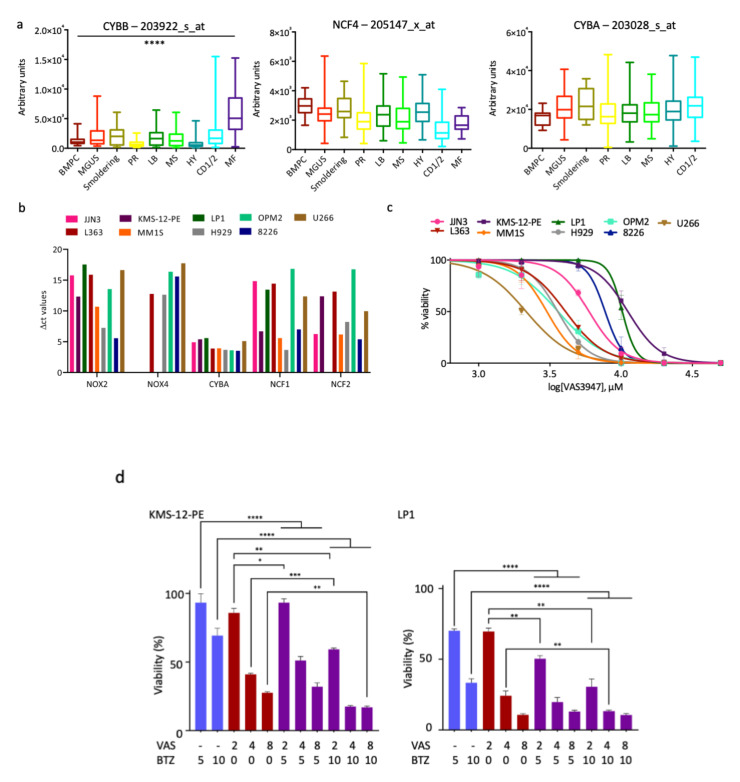
VAS3947 induces MM cell death but could have adverse effects when combined with bortezomib. (**a**) We used the Amazonia! tool (http://amazonia.transcriptome.eu/) for GEP analysis of *CYBB* (NOX2), *NCF4* (p40^phox^), and *CYBA* (p22^phox^) genes from Zhan datasets [21]. The expression signal of the indicated probes in arbitrary units was presented as boxplots for BMPC (*n* = 22), MGUS patients (*n* = 44), patients with smouldering MM (*n* = 12), or overt MM (*n* = 414). MM patients were classified at diagnosis according to their molecular subgroups: PR (*n* = 47), LB (*n* = 58), MS (*n* = 68), HY (*n* = 116), CD1/2 (*n* = 88), MF (*n* = 37). **** *p* < 0.0001 with the Mann-Whitney test; (**b**) The level of NOX subunits gene expression of the different MM cell lines was compared by a semi-quantitative RT-PCR. The results were normalized to housekeeping genes (*GAPDH*, *ACTB* and *RPL13A*) and presented as ΔCt = “Ct target” − “Ct reference” (in triplicate, data are expressed as the means). *NOX1* is expressed only in L363 and U266 cells, *DUOX1* in MM.1S and U266 cells whereas *NOX3* is not expressed (Table S5); (**c**) The indicated nine MM cell lines were assayed for their sensitivity towards VAS. Cells were seeded in 96-well plates at a density of 5 × 10^4^ cells/well and treated with vehicle or increasing concentrations of VAS (1–50 μM) for 48 h. The viability of each cell line treated with the drug, determined by an MTS assay, is expressed relative to that of the cell line treated with the vehicle (0.01% DMSO, defined as 100%). For each culture condition, the mean of triplicate ratios is indicated on the graph, together with the SD. Three independent experiments have been performed. The curves have been drawn with the Prism software and the calculated IC_50_ are reported in Table 1; (**d**) KMS-12-PE and LP1 cells (both little sensitive to BTZ) were assayed for VAS sensitivity alone and VAS/BTZ combination with the indicated concentrations. Cells were treated with drugs or vehicle (0.01% DMSO) for 24 h and their viability assessed as before with the MTS assay. Bar graphs correspond to the means + SD of triplicate ratios. The experiment has been performed twice. * *p* < 0.05, ** *p* < 0.01, *** *p* < 0.001, and **** *p* < 0.0001 with the *t*-test for two groups or the ANOVA test for multiple groups.

We interrogated publicly available microarray datasets [21] and analysed NOX complexes subunits (NOX1-5, DUOX1/2, NCF1 or p47^phox^, NCF2 or p67^phox^, CYBA or p22^phox^, NCF4 or p40^phox^, NOXA1 and NOXO1) expression (GEP, Table S4). We compared normal bone marrow plasma cells (BMPC), patients with a monoclonal gammopathy of undetermined significance (MGUS), smouldering myeloma or overt myeloma at diagnosis. MM patients were categorised according to their molecular subgroups, defined by Zhan and coworkers [21] as proliferating (PR), low bone disease (LB), with activated FGFR3/MMSET genes (MS), hyperdiploid (HY), expressing cyclin D1 (CD1/2), with activated MAF gene (MF). NOX2 (CYBB) was the only catalytic subunit expressed in all MM patients and, compared with BMPC samples, overexpressed in the MF group (Figure 1a). Among the regulatory subunits, only NCF4 (p40^phox^) and CYBA (p22^phox^) were transcribed in all patients (Figure 1a).

We next analysed and quantified the expression of the same NOX subunits in a panel of MM cell lines according to a previously published method of semi-quantitative RT-PCR [13]. The level of each gene was expressed as ΔCt values that were normalized with three internal reference genes (*GAPDH*, *RPL13A*, *ACTB*) (Table S3). In good agreement with data obtained for MM patients, MM cell lines expressed homogenously NOX2 (CYBB) as catalytic subunit as well as CYBA (p22^phox^), NCF1 (p47^phox^), and NCF2 (p67^phox^) as regulatory subunits (Figure 1b, Table S5).

### 3.2. A Pan-NOX Inhibitor Is Cytotoxic on MM Cell Lines but Has Adverse Effects When Combined with Bortezomib

ROS are constitutively produced in MM cells (Figure S1b) and, at least in part, by the NOX complexes activity [13]. Since manipulating cellular redox parameters is a potent way to trigger apoptosis [15], we inhibited NOX2/4 to decrease ROS levels and disturb redox homeostasis. We used VAS, a pan-NOX inhibitor and assessed MM cell viability with an MTS test. The results are reported in Figure 1c. Among the MM cell lines tested, some are highly sensitive (L363, MM.1S) whereas others, including KMS-12-PE and LP1, are less sensitive. This was confirmed by the calculation of the index of cytotoxicity (IC_50_) after a 48 h-treatment (Table 1). We previously determined that, compared to other cell lines, KMS-12-PE and LP1 cell lines were also less sensitive to BTZ (Figure S1c, Table 1). We then analysed the combination VAS/BTZ. For that purpose, we treated KMS-12-PE and LP1 cells with VAS at concentrations lower or equal to the IC_50_ (2–8 μM) alone or with BTZ (5 or 10 nM). Cell viability was assessed by the MTS assay; the results are reported in Figure 1d. We observed that the response to VAS and BTZ was dose-dependent in KMS-12-PE and LP1 cells. Moreover, compared to the treatment with BTZ alone, for the highest tested doses (VAS 8 μM and BTZ 10 nM), the VAS/BTZ combination decreased cell viability by 56.07% and 24.95%, in KMS-12-PE and LP1 cells, respectively (Figure 1d). However, although VAS significantly increased BTZ anti-MM activity, the Chou-Talalay combination index indicated either an additive effect for some combinations or an antagonistic one for others (Table 2). These data exclude the use of VAS as an anti-MM therapy.

### 3.3. Antioxidant Proteins Are Overexpressed in Myeloma Patients and MM Cell Lines

**Figure 2:**
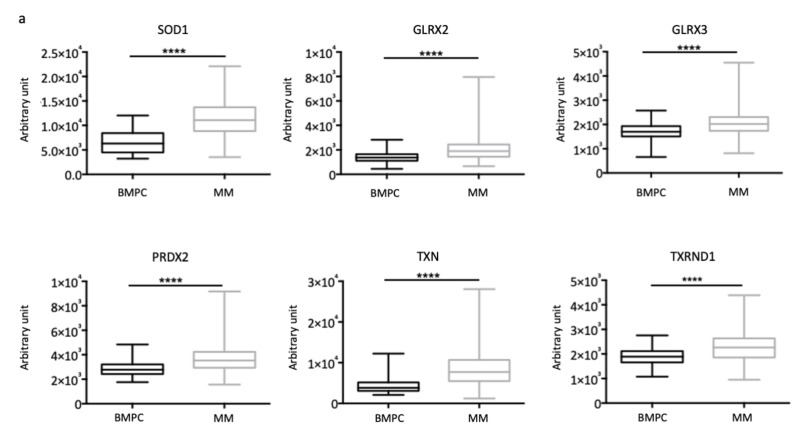

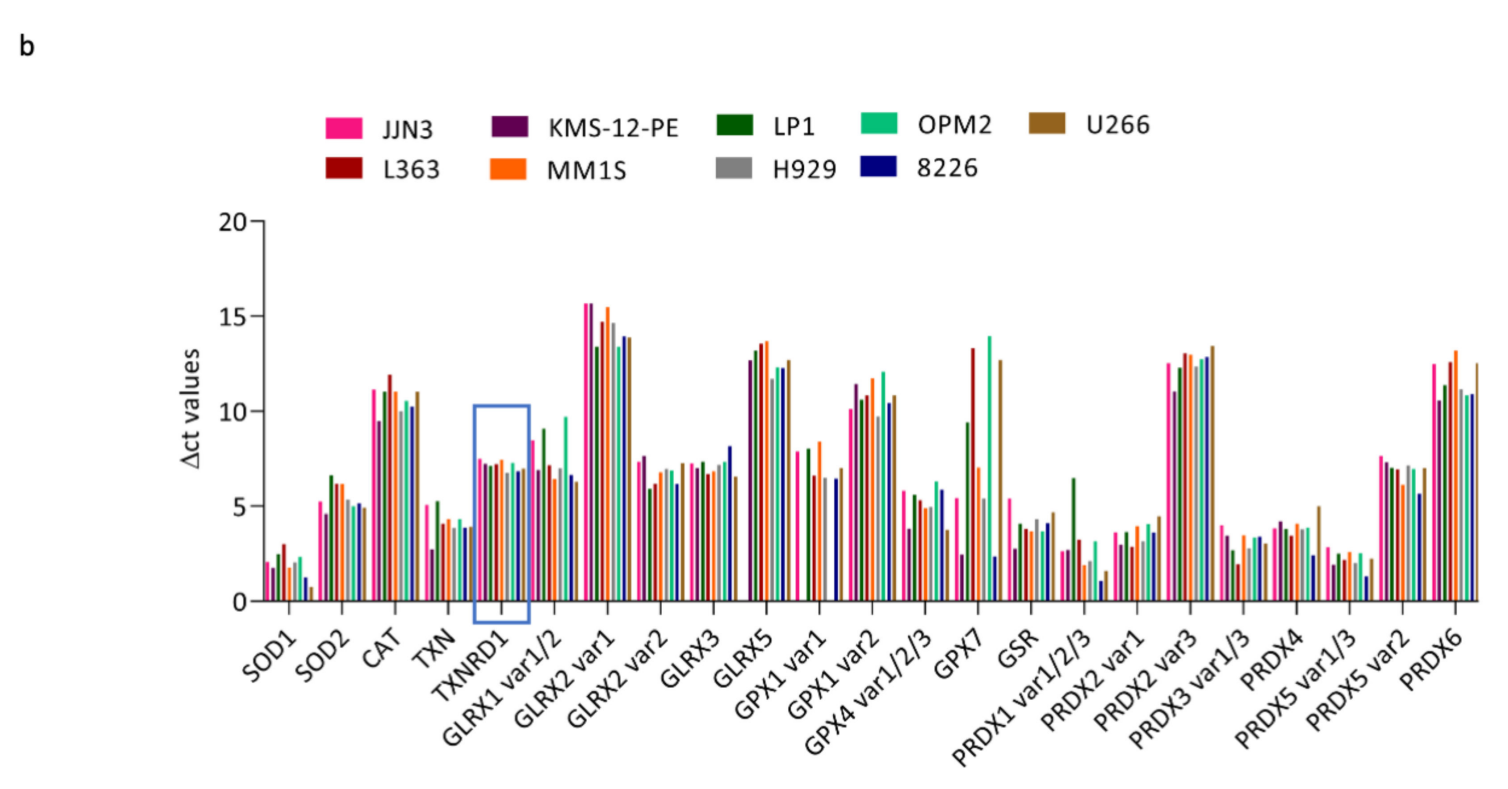
Antioxidant enzymes are overexpressed in MM patients and cell lines. (**a**) We used the Amazonia! tool (http://amazonia.transcriptome.eu/) for GEP analysis of *SOD1*, *GLRX2/3*, *PRDX6*, *TXN*, and *TXNRD1* within Zhan datasets [21]. The expression signal of the indicated probes in arbitrary units was presented as boxplots for BMPC (*n* = 22) or overt MM (*n* = 414). **** *p* < 0.0001 with the Mann-Whitney test; (**b**) The expression level of genes coding for antioxidant enzymes was compared by a semi-quantitative RT-PCR in the various MM cell lines. The results were normalized to housekeeping genes (*GAPDH*, *ACTB*, and *RPL13A*) and presented as ΔCt = “Ct target” − “Ct reference” (in triplicate, data are expressed as the means). *GPX2* is expressed only in MM.1S, *GPX3* in JJN3 cells; *SOD3* is not expressed (Table S6).

At least for some concentrations of the NOX inhibitor, the decrease of ROS showed antagonistic effects when associated with BTZ, which enhances ROS production. We next investigated whether a molecule that overproduces ROS could have opposite effects. To do so, we choose to target antioxidant enzymes that defend cells against drug-induced oxidative stress. We first interrogated publicly available microarray datasets [21] and analysed detoxifying enzymes GEP (Table S4). We compared normal BMPC and patients with overt myeloma at diagnosis. As shown in Figure 2a, *TXN* and *TXNRD1* were overexpressed in MM patients confirming previous data [22,23]. Moreover, highlighting our hypothesis, thioredoxin (TXN) inhibition overcomes BTZ-resistance in MM cells [23] and TXN and thioredoxin reductase 1 (TXNRD1) overexpression was also associated with BTZ resistance in a comparative proteomic profiling [12]. *SOD1*, *GLRX2*, *PRDX6*, and *GLRX3* genes were also overexpressed in MM patients compared to normal plasma cells, confirming that the redox status is unbalanced in tumour vs. normal cells (Figure 2a). We next analysed the correlation of detoxifying enzymes with molecular subtypes of MM patients and observed some differences among the subgroups (Figure S2). For example, *GLRX2* and *GLRX3* were overexpressed in three groups (PR, MS, and MF, *p* < 0.0001) with an adverse prognosis and *TXN* was overexpressed in the PR molecular subgroup (*p* < 0.0001). Moreover, high levels of *PRDX6* or *SOD1* were associated with a shorter overall survival among MM patients, whereas the expression of *GLRX3*, *GLRX2*, *TXN*, and *TXNRD1* did not show any correlation (Figure S3). Thus, the targeting of antioxidant enzymes may be relevant for anti-myeloma therapy, including all molecular sub-groups.

We also characterised the MM cell lines regarding the expression of the same antioxidant enzymes by a semi-quantitative RT-PCR. The results are reported in Figure 2b and Table S6. Although MM cell lines belong to various molecular sub-groups (Table S1), some genes encoding antioxidant enzymes were highly and homogenously expressed including *SOD1*, *TXN*, *GLRX2/3*, and *PRDX2* (Figure 2b). Importantly, the *TXNRD1* gene (squared in blue) was expressed in the panel of MM cells, as well as in MM patients (Figure 2a,b).

### 3.4. A Thioredoxine Reductase Inhibitor Is Cytotoxic on MM Cell Lines and Has Synergistic Effects When Combined with Bortezomib

**Figure 3:**
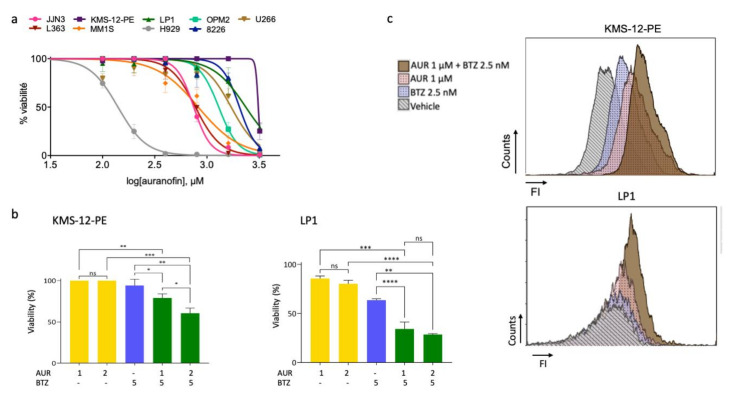
Auranofin and bortezomib act synergistically and generate ROS in MM cells. (**a**) The nine indicated MM cell lines were assayed for their sensitivity/resistance towards AUR. The cells were seeded in 96-well plates at a density of 5 × 10^4^ cells/well and treated with vehicle (0.01% DMSO) or increasing concentrations of AUR (0.1–3.2 μM) for 48 h. The viability of each cell line treated with the drug, determined by an MTS assay, is expressed relative to that of the cell line treated with the vehicle (defined as 100%). For each culture condition, the means of triplicate ratios are indicated on the graph, together with the SD. Three independent experiments have been done. The curves have been drawn with the Prism software and the calculated IC_50_ are reported in Table 1; (**b**) KMS-12-PE and LP1 cells were assayed for AUR-sensitivity alone (A, 1–2 μM), BTZ- (B, 5 nM) or AUR/BTZ combination with an MTS assay after a 24-h period. Bar graphs correspond to the means + SD of triplicate ratios. The experiment has been performed three times; a representative one is shown. ns, not significant; * *p* < 0.05, ** *p* < 0.01, and *** *p* < 0.001 by the *t*-test; (**c**) KMS-12-PE and LP1 cells were treated with vehicle (grey), 2.5 nM BTZ (blue), 1 μM AUR (red) or combo (brown) for 24 h, then stained with the CellROX Deep Red fluorescent probe and analysed with the NucleoCounter NC-3000. The fluorescence intensity (FI) of each culture condition is represented in the *X*-axis, and the number of counts in the *Y*-axis. The experiments has been performed three times for KMS-12-PE cells, twice for LP1 cells; a representative one is shown.

TXNRD1 along with TXN and NADPH belongs to the major disulfide reductase antioxidant system in humans, thereby maintaining the redox homeostasis. We used the gold complex auranofin (AUR) as an inhibitor of TXNRD1. AUR induces a cytotoxic ER stress and exerts a cytotoxic activity against B-cell chronic lymphocytic leukaemia, B-cell lymphomas and MM cells [24,25,26,27]. We analysed the antitumour effects of AUR (0.1–3.2 μM) on the same nine MM cell lines after a 48-h treatment. As reported in Figure 3a and Table 1, MM cells displayed various sensitivity according to their calculated IC_50_. H929, JJN3, L363, and MM.1S were the most sensitive cells whereas KMS-12-PE, LP1, and 8228 were the less sensitive. We next analysed in the same settings, AUR and BTZ single-treatments, and the combined BTZ/AUR treatment (Figure 3b, Table 3, Figure S1d). BTZ/AUR combination treatments showed substantial synergistic effects on MM cell lines (Table 3). Importantly, AUR treatment enhanced BTZ-sensitivity of KMS-12-PE and LP1 cells (Table 1 and Table 3). As visualised by CellROX Deep Red staining, the production of ROS that occurred after BTZ or AUR single treatments was increased by the combination in KMS-12-PE and LP1 cells (Figure 3c).

### 3.5. Bortezomib/Auranofin Combined Treatment Triggers Apoptosis and Auranofin Activates Autophagy

**Figure 4:**
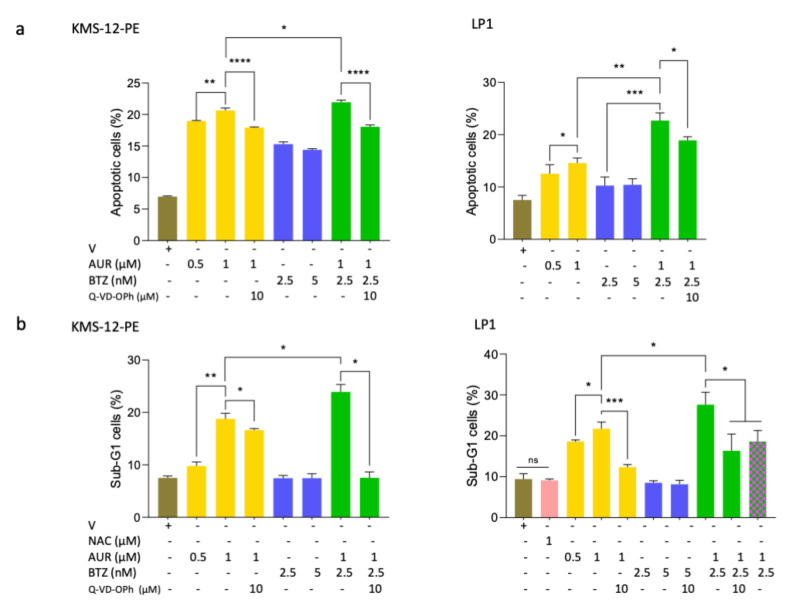
Auranofin induces apoptosis in MM cells. (**a**) KMS-12-PE and LP1 BTZ-resistant cells were treated for 24 h with vehicle (V), AUR (0.5–1 μM), BTZ (2.5–5 nM), or AUR/BTZ combo and assessed thereafter. For the inhibition of caspases, cells were treated with 10 μM Q-VD-OPh for 1 h before drugs treatment. Cells were then stained with Hoechst 33342, PI and annexin V-FITC for 30 min at 37 °C and immediately analysed with the NucleoCounter NC-3000. Data obtained from the NucleoView software were exported into the Prism software to draw the histograms that show the means ± SD of the percentage of annexin V-positive cells. Three (LP1) or four (KMS-12-PE) independent experiments in triplicate have been done, a representative one is shown; (**b**) KMS-12-PE and LP1 cells were treated as described in (**a**). Moreover, LP1 cells were also treated with 1 mM NAC for 12 h and then with AUR/BTZ combo. Treated-cells were fixed in EtOH and incubated in a buffer containing RNase A and DAPI. Cell cycle was analysed by image cytometry (NucleoCounter NC-3000, ChemoMetec). At least 10^4^ cells were analysed for each culture condition. The number of cells in each phase of the cell cycle (sub-G1, G0/G1, S, G2/M) was determined by the Kaluza software (Beckman Coulter). Data were exported and analysed with the Prism software. Histograms representing the means ± SD of the percentage of apoptotic cells were drawn with Prism and the *p*-values calculated by the same software with the *t*-test. The experiment has been done three times with triplicate samples for each cell line, a representative experiment is shown; * *p* < 0.05; ** *p* < 0.01; *** *p* < 0.001; **** *p* < 0.0001 with the *t*-test.

To examine the molecular basis of BTZ/AUR sensitivity, we analysed KMS-12-PE and LP1 cells for apoptosis triggering. Cultured cells were treated with vehicle, BTZ (2.5–5 nM), AUR (0.5–1 μM), or combination drugs for 24 h and analysed for annexin V/PI staining using image cytometry. Compared to the vehicle, the AUR-single treatment induced a significant percentage of annexin V-positive i.e., apoptotic cells (+13.6% and +7.1%, for the highest concentration tested, in KMS-12-PE and LP1 cells, respectively) (Figure 4a). Compared to the single BTZ-treatment, the combination increased this percentage (+6.6% and +12.4% for KMS-12-PE and LP1, respectively) (Figure 4a). In both cell lines, the treatment with the Q-VD-OPh pan-caspase inhibitor completely reversed the apoptotic response induced by the single AUR-treatment and the combination confirming that AUR signalled through a caspase-dependent apoptosis (Figure 4a).

We confirmed the induction of a caspase-dependent apoptosis by the analysis of cell cycle distribution of treated cells. Indeed, the appearance of a cells having a sub-G1 DNA content is reminiscent of DNA degradation, a late stage of apoptosis. As before, compared to vehicle, the single AUR-treatment increased the percentage of apoptotic cells in both cell lines (+10.7% and +9.2% for KMS-12-PE and LP1, respectively) (Figure 4b). Moreover, compared to the single BTZ-treatment, the combination increased this percentage (+16.4% and +19.1% for KMS-12-PE and LP1, respectively) (Figure 4b). The Q-VD-OPh-treatment inhibited the apoptosis induction as well as the NAC-treatment in LP1 cells (Figure 4b). Using the same assay, we verified that AUR and combination treatments activated a caspase-dependent apoptosis also the BTZ-sensitive L363 cells. Moreover, in these cells, the triggering of apoptosis was inhibited by the NAC-treatment (Figure S4).

**Figure 5:**
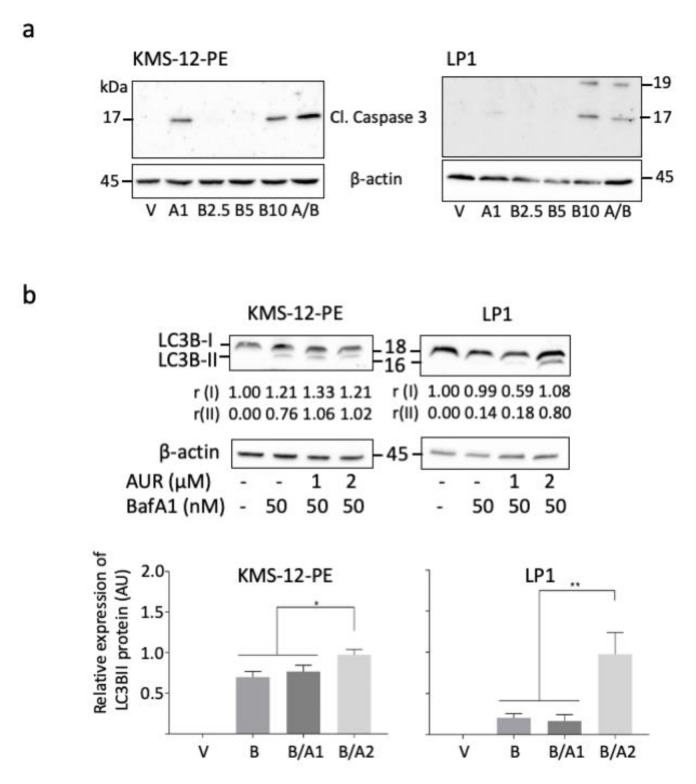
Auranofin induces a caspase-dependent apoptosis and autophagy in MM cells. (**a**) KMS-12-PE and LP1 cells were treated with vehicle (V), 1 μM AUR (A1), 2.5–10 nM BTZ (B2.5–B10), or 1 μM AUR plus 2.5 nM BTZ (A/B) for 24 h. Whole-cell protein extracts were prepared and separated by SDS-PAGE. Proteins were blotted and analysed with an anti-cleaved (Cl.) caspase 3 Ab (#9664 from Cell Signaling Technologies). The Ab detects two forms of 19 and 17 kDa. An anti-β-actin Ab was used as a control of loading and transfer. These results were confirmed with a new series of blots in Figure S5. The estimation of each protein level is presented in Table S7; (**b**) KMS-12-PE and LP1 cells were treated with vehicle (V) or 1–2 μM AUR for 24 h (or not for a control) and then with 50 nM BafA1 for 4 h. Whole-cell protein extracts were obtained, separated by SDS-PAGE, and transferred onto membranes. IB were incubated with an anti-LC3B Ab and an anti-β-actin Ab as a control. The level of LC3B-I and -II forms was estimated by densitometry and normalised against the β-actin level (rI and rII, respectively). Data collected from three independent experiments (Figure S5) were used to draw the histograms (means ± SD). * *p* < 0.05 and ** *p* < 0.01 with an ANOVA test.

We analysed the downstream effects of apoptosis induction by appearance of cleaved activated caspase 3. Indeed, as observed Figure 5a, the cleaved form of caspase 3 was detected after a 1 μM-treatment for both cells. The intensity of the band increased when AUR (1 μM) and BTZ (2.5 nM) were combined (r = 1.64 vs. 1.10 and 2.64/2.25 vs. 1.00/1.00 for KMS-12-PE and LP1, respectively, Table S7). These data were confirmed with a new series of IB obtained with similar cells extracts but a new Ab against cleaved caspase 3 (Figure S5a). We concluded that in MM cells, apoptosis proceeded through the intrinsec mitochondrial caspase-dependent pathway.

The generation of ROS induces a protective mechanism of autophagy in MM cells [27]. Conversely, if the UPR is chronically compromised, autophagy results in cell death [28]. The microtubule-associated protein 1A/B-light chain 3B (or LC3B) is translocated to autophagosomes and cleaved (LC3B-I to LC3B-II) at the onset of autophagy. In turn, an increase of LC3B-II level is a reliable marker of autophagy. The conversion LC3B-I to LC3B-II triggered by a bafilomycin A1 (BafA1) was enhanced by an AUR-treatment (1 or 2 μM), indicating that AUR induced an autophagic flux in MM cells (Figure 5b). This was evidenced in both KMS-12-PE and LP1 cells, although the AUR dose-effect was observed only for LP1 cells (Figure 5b). As observed previously, KMS-12-PE cells are less sensitive to AUR-treatments than LP1 cells (Figure 3 and Figure 4). As a whole, the death of MM cells after an AUR-treatment proceeds through both apoptotic and autophagic mechanisms.

### 3.6. Bortezomib/Auranofin Combined Treatment Alleviates TME-Mediated Cell Resistance

We previously reported that MM cells cultured on FBN-coated plates exhibited a marked resistance towards CFZ [13]. Indeed, MM cells interact with FBN trough their integrin receptors such as they do in their TME. In turn, a cell adhesion-mediated drug resistance (CAM-DR) phenomenon occurs. We confirmed this observation by quantifying MM cell death after AUR-, BTZ- or combined treatments in H929 and L363 cells when cultured either in suspension or on FBN (Figure S6a,b). For each drug treatment, we calculated an index of resistance (RI) corresponding to the viability of treated cells in FBN-coated plates relative to the viability of cells cultured in suspension (Figure S6c). L363 cells were particularly sensitive to AUR-treatment even when cultured on FBN (viability of 31.4% and 41.2%, respectively). However, the AUR/BTZ combination was more potent for inducing cell death by each drug alone in both culture conditions (−12.1% and −8.4% for viability, for the lowest concentration of AUR, and cells cultured in suspension vs. on FBN-coated plates). In contrast, a clear CAM-DR was observed for H929 cells treated with AUR alone or BTZ alone (+ 20.6% and +12.0% of viability, respectively). Again, the combination AUR/BTZ significantly decreased CAM-DR (−17.7% and −29.9% for viability, for the lowest concentration of AUR, comparing cells in suspension vs. on FBN-coated plates). These data suggest that AUR indirectly modified MM cells/microenvironment interactions. In the same experimental conditions, the death of LP1 and KMS-12-PE was almost impossible to induce (Figure S7).

**Figure 6:**
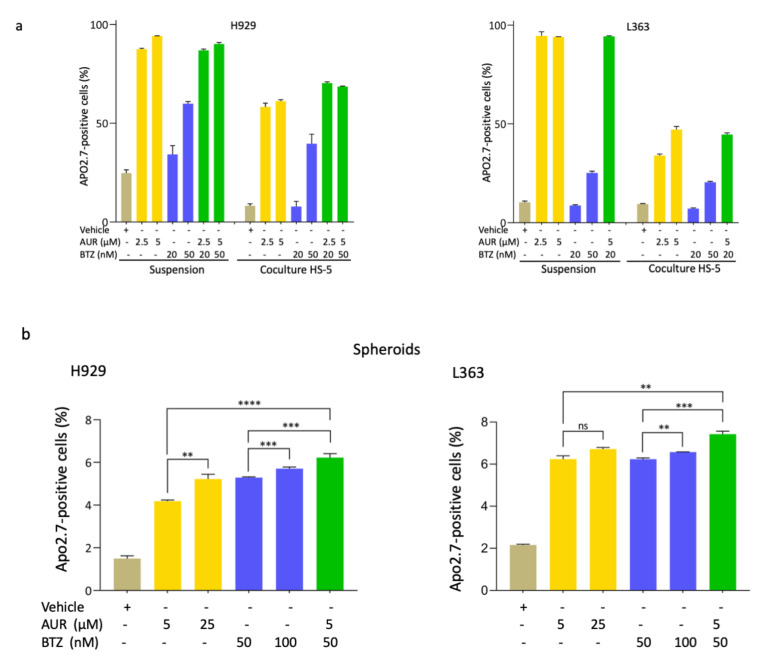
AUR/BTZ combined treatment alleviates CAM-DR. (**a**) H929 and L363 BTZ-sensitive cells were seeded at a density of 10^5^ cells/well, then cultured in suspension or in co-culture with HS-5 mesenchymal cells. Cells were treated 1 h later with AUR (μM), BTZ (nM) or combo for 24 h at the indicated doses. Apoptosis induction was quantified after cell staining with a PE-conjugated anti-APO2.7 Ab (cells in suspension) and co-staining with a APC-conjugated anti-CD10 Ab (coculture), then analysed by flow cytometry. The number of APO2.7-positive and CD10-negative cells (coculture) was recorded and used to draw the histograms with the Prism software. The histograms are the means ± SD from one indicative experiment performed with triplicate samples out of three independent experiments performed. The *p*-values for the comparison between the treatments or the culture models calculated with the *t*-test are indicated in Table 4 and Table 5; (**b**) H929 and L363 cells were seeded and cultured in a 3-D reconstructed bone marrow model. Briefly, MM cells were mixed in a matrix containing FBN, collagen I and IV, and matrigel and cultured in complete medium in 24-well plates. Spheroids formed as soon as two days after seeding. Six days later, cultures were treated with drugs and combo at the indicated doses for 48 h. Then, cells were dissociated with a mixture of EDTA, Na_3_VO_4_ and NaF, and assayed after APO2.7 staining as described in (**a**). The histograms drawn with the Prism software are means ± SD from one representative experiment performed in triplicate. The experiment was repeated three times. ns, not significant; ** *p* < 0.01; *** *p* < 0.001, and **** *p* < 0.0001 with the *t*-test.

As another more complex TME model, we tested the response of H929 and L363 BTZ-sensitive cells cultured on a layer of HS-5 bone marrow mesenchymal cells, mimicking the bone marrow environment and also allowing CAM-DR [13]. L363 and H929 BTZ-sensitive cells were treated with AUR, BTZ, or AUR/BTZ combination. For each single treatment and combination, for the two cell lines, we observed a correlation between the number of apoptotic cells (APO2.7-positive) and the concentration of the drug (Figure 6a). This dose-response effect was confirmed statistically (Table 4). As expected from our previous results, the AUR/BTZ combination, although more potent than the single treatment, was less efficient in cells cultured in coculture (Figure 6a, Table 5, Table S8). For the combination 2.5 μM AUR plus 20 nM BTZ, compared to the 20 nM dose of BTZ, we observed an increase of 56.7% and 60.7% of apoptotic cells for H929 cells cultured in suspension vs. on HS-5 cells, respectively, and 85.4% and 45.2% for L363 cells in the same settings. The statistical analyses are reported in Table 4 and Table 5. Importantly, the calculated Chou-Talalay CI confirmed that the AUR/BTZ combination acted synergistically in both cell lines and in both culture models (Table 6).

We next used a 3-D reconstructed bone marrow-based culture model in which either MM cell lines or primary cells grew in spheroids, mimicking the “real-life” (Figure S8a–d). As soon as two days after seeding, in the 3-D model, MM cells aggregated in small clusters. These clusters grew and six days after seeding, they were composed of an average of 7–8 cells for the three cell lines assayed (L363, H929 or LP1) or 20–30 cells for MM Pt #1.

MM cell lines cultured in 3-D acquired a resistant phenotype as observed by the decrease of apoptotic (APO2.7-positive) cells when treated with AUR, BTZ-single treatments (−90.0% (AUR), and −45.6% (BTZ) apoptotic cells for the comparison of suspension vs. spheroid for H929 cells; and −87.8% (AUR) and −18.9% (BTZ) for L363 cells) (Figure 6b, Table S8). Importantly, the combination AUR/BTZ was still efficient in synergy, even when H929 and L363 cells were cultured in spheroids (Table 6, Table S8).

### 3.7. Auranofin Triggers Apoptosis of Primary MM Cells

**Figure 7:**
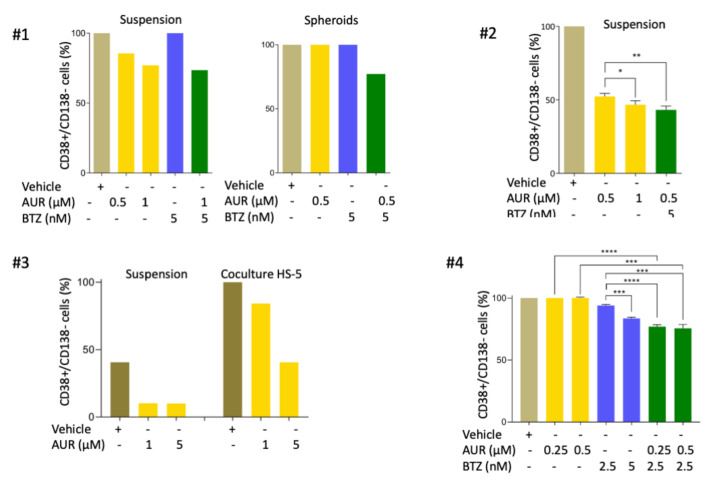
Auranofin induces apoptosis in primary cells of MM patients and co-operates with BTZ to restore sensitivity. Mononuclear cells were purified from bone marrow cells of MM patients at diagnosis. Cells were cultured in complete medium supplemented with rIL6 in suspension (Pts #1, 2 and 3), on a layer of HS-5 cells (Pts #3 and 4), or in spheroids (Pt #1). Cells were then treated with AUR (0.5–1 μM), BTZ (2.5–5 nM) or when possible in AUR/BTZ combination (Pts #1, 2, and 4). For Pts #2 and #4, we had enough tumour cells to test the various culture conditions in triplicate. The induction of MM apoptosis was assayed by the loss of CD138; the histograms present the number of CD38-positive/CD138-negative cells after a 24-h treatment. The means ± SD are indicated on the graph for Pts #2 and 4, as well as the *p*-value obtained with the *t*-test. * *p* < 0.05; ** *p* < 0.01; *** *p* < 0.001.

We finally analysed the response of primary cells isolated from four MM patients (Pt #1-4, Table S2) towards AUR, BTZ, and the AUR/BTZ combination when possible. Primary MM cells were cultured either in suspension (#1-4), in coculture with HS-5 cells (#3 and 4), or in spheroids (#1). In these settings, the effect of drugs was monitored by the decrease of the CD138-positive fraction after CD38/CD138 costaining [18]. We first observed a dose-response of primary cells to AUR single-treatment for cells cultured in suspension (Pts #1, 2) and in co-culture with HS-5 (Pt #3) (Figure 7). In contrast, the Pt #4 is more sensitive to BTZ than to AUR. Although the number of patients was limited, they recapitulated the diversity of response observed with the cell lines. Importantly, compared to BTZ alone (Pts #1, 2 and 3) or to AUR alone (Pt #4), the AUR/BTZ combination enhanced the number of CD138-positive cells’ decrease. Indeed, compared to AUR alone, the BTZ/AUR combination increased the percentage of apoptotic cells by 26.4% and 9.2% in cells cultured in suspension, for Pts #1 and 2, respectively. For MM cells cultured in coculture with HS-5 (Pt #4), upon the combination treatment, the increase was found to be 18.3% and 25.0%, compared to BTZ alone and AUR alone, respectively. Finally, in the 3-D spheroid model (Pt #1), the AUR/BTZ combination increased apoptosis by 22.8% compared to both AUR and BTZ single-treatments. Our data revealed at least an additive effect of the AUR/BTZ combination in primary cells.

## 4. Discussion

In eukaryotic cells including cancer cells, ROS are generated by various endogenous and exogenous sources during various cell processes, including oxidative metabolism. To maintain ROS at physiological concentrations, their production and availability are finely regulated [29]. In cancer cells, the level of ROS determines their effects. When present at low or intermediate levels, ROS control proliferation, invasion, angiogenesis, and drug response. At high levels, ROS induce irreversible DNA damage and ultimately cell death [30]. Due to their hypoxic environment in the bone marrow, MM cells produce high levels of ROS but are adapted to this generated chronic oxidative stress. Nevertheless, the current standards of care for newly diagnosed MM are BTZ-containing regimens [31] and BTZ-mediated cell death is due, at least in part, to the production of intracellular ROS [32]. Our findings confirmed that any modification of the balance between ROS production and scavenging triggers MM cell death.

We first inhibited ROS production by inhibiting the NADPH oxidase NOX2. NOX2 is the sole catalytic subunit of NADPH oxidase expressed in MM cells (Figure 1a) and produces ROS as its main function [33]. The inhibition of NOX2 with VAS3947, a pan-NOX inhibitor, has a cytotoxic effect, at least in responsive cells (Figure 1c). However, its association with BTZ appears to be inefficient and could even be deleterious for MM patients (Figure 1d, Table 1 and Table 2). Since the inhibition of ROS production does not show the expected effects, we decided to modify the redox equilibrium by increasing ROS production. We successfully did that by inhibiting engagement of detoxifying factors. We report that AUR, an inhibitor of TXNRD1, acts in synergy with BTZ in sensitive and insensitive cell lines and primary cells to induce MM cell death (Table 3, Figure 3, Figure 4, Figure 5 and Figure 6). Moreover, AUR/BTZ combination alleviates BTZ-resistance in several in vitro settings (Figure 6, Figures S6 and S7). Indeed, AUR/BTZ was efficient on MM cells and primary cells cultured on a layer of HS-5 stromal cells and in a 3-D model mimicking the bone marrow niche (Figure 6a,b and Figure 7). The interactions of malignant MM cells with TME facilitate a drug-resistant phenotype [34]. We observed that it was obviously the case when cells were cultured on FBN-coated plates, in coculture with mesenchymal cells, or in spheroids (Figure 6 and Figure 7; Figures S6 and S7). In turn, studies in such models could be more representative of the response of MM cells towards drugs. Although the efficacy of AUR has been reported previously for MM cell lines and patients [26,35], its effect on TME-induced resistance to BTZ was not. Therefore, we propose considering the combination AUR/BTZ for refractory and relapse (R/R) MM patients. Importantly, the combination is effective in MM cells having a distinct genetic background and in particular, exhibiting either an abnormal or a wild-type *TP53* status (e.g., H929 vs. L363 cell lines, Table S1); this is an important point since MM cells frequently exhibit an inactivation of the p53 pathway [35]. The anti-MM activity of AUR varies among cell lines (Table 1) and MM patients (Figure 7) likely because they possess intrinsic differential sensitivities to oxidative stress and antioxidant capacities.

We observed that *TXN* and *TXNRD1* coding for antioxidant enzymes are both overexpressed in MM compared to normal plasma cells (Figure 2) thereby confirming a previous report [26]. Furthermore, we provided evidence that other antioxidant enzymes such as [Cu-Zn] superoxide dismutase (SOD1), glutaredoxin 2/3 (GLRX2/3), and peroxiredoxin 6 (PRDX6) are also overexpressed (Figure 2). Moreover, according to the molecular classification of MM [21], the proliferating (PR) molecular subtype that is associated with a poor prognosis and a bad response to treatments, is characterized by an overexpression of *GLRX2/3* and *PRDX6* mRNAs (Figure S2). Although the inhibition of TXNRD1 alone overcomes BTZ-resistance, cross-talk between other detoxifying enzymes may occur, in turn, limiting cell death. To our knowledge, the effects of GLRX2/3 and PRDX6 inhibitors in MM have not been reported so far. However, interestingly, the pharmacological inhibition of SOD1 by disulfiram enhances BTZ toxicity [36]. As a whole, antioxidant enzymes may be considered as potent targets for R/R patients and the targeting of more than one enzyme may be a suitable option.

Mechanistic studies showed that AUR produces ROS (Figure 3c) and that ROS trigger apoptosis through the activation of executioner caspase 3 (Figure 5a). More interestingly, besides this apoptotic component, cell death proceeds also through autophagy (Figure 5b). Zheng et al. reported recently that PX12, an inhibitor of TXN, induces mitophagy in MM cells [23]. Mitophagy is an autophagic process of mitochondria capture and degradation by lysosomes. Moreover, the process of autophagy seems a general response to AUR or more generally to TXNRD1 inhibition in solid cancers [37,38,39]. MM cells synthesize large amounts of immunoglobulins and autophagy is a process by which MM cells protect themselves from unfolded or misfolded proteins. Compromising the UPR cascade induces an autophagic cell death in MM cells [29]. Our data confirm that ROS overproduction and subsequent redox unbalance also trigger an autophagic cell death in MM.

Current MM treatments involves proteasome inhibitors, immunomodulators, an alkylating agent melphalan, and high-dose dexamethasone [1]. Gourzones et al. reported that melphalan induces ROS and antioxidants involved in glutathione synthesis and regeneration protect cells from melphalan-induced cell death [40]. However, other antioxidant drugs have no effect on melphalan-mediated toxicity while protecting cells from BTZ. These data underline that all ROS-producing agents do not target the same systems, complexifying our understanding of the redox control. Since MM cells are particularly responsive to ROS inducers agents, a better knowledge of this redox control is essential for adapting new therapeutic protocols. This point is reinforced by the observation that the level of mRNA of two antioxidant genes *PRDX6* and *SOD1* are directly correlated with the overall survival of MM patients at diagnosis (Figure S3).

MM cells show complex redox and energy metabolism changes that account for chemoresistance [41]. However, we successfully induce MM cell death and alleviate chemoresistance by combining BTZ that induces an oxidative stress, and AUR that inhibits the TXNRD1 antioxidant enzyme and further perturbs the redox balance. This was achieved in in vitro models mimicking the tumour niche. AUR is FDA-approved for the treatment of rheumatoid arthritis and is currently assessed in clinical trials for ovarian cancers, lung cancers, and chronic lymphocytic leukaemia (ClinicalTrials.gov). Since AUR is selectively toxic for MM cells and spares CD34+ progenitors, B cells [24] and normal plasma cells [26], our preclinical findings open new perspectives for R/R MM patients. Importantly, the efficiency of CFZ, a new-generation proteasome inhibitor, which seems more potent for MM patients, is enhanced when associated with resveratrol that generates ROS [42].

To conclude, BTZ generates ER stress and activates the UPR pathway, and, in turn, increases the level of intracellular ROS in myeloma cells. However, the unbalance of ROS levels through UPR activation with BTZ and inhibition of NOX with VAS, although both cytotoxic on myeloma cells as single agents, could have deleterious effects when combined. In contrast, the overproduction of ROS by the inhibition of the TXNRD1 antioxidant enzyme with AUR, on one hand, and the activation of UPR with BTZ, on the other, could efficiently be combined. This association is potent on MM cell lines, primary MM cells and reverses, at least in part, TME-mediated drug resistance.

## Figures and Tables

**Table 1:** Sensitivity of MM cell lines towards the drugs used.

Cell Lines	IC_50_ VAS (μM)	IC_50_ AUR (μM)	IC_50_ BTZ (nM)
JJN3	>4.3	0.8	4.6
KMS-12-PE	7.8	>3.5	19.2
LP1	8.0	>3.0	9.8
L363	3.7	0.9	4.3
MM.1S	1.5	0.4	nd
H929	2.7	0.09	3.7
OPM2	3.2	1.2	2.7
8226	6.4	>4.3	9.1
U266	2.6	2.2	4.8

MM cell lines were seeded in 96-well plates at a density of 5 × 10^4^ cells per well, then treated for 48 h with vehicle, VAS (1–50 μM), AUR (100 nM–5 μM), or BTZ (1–30 nM). Cell viability was assayed using an MTS assay (CellTiter 96^®^ AQueous One Solution Cell Proliferation Assay, Promega). The cell viability in each culture condition in triplicate was determined. The Prism software was used to calculate the corresponding IC_50_ and the calculated IC_50_ was verified with the CompuSyn software. nd, not done.

**Table 2:** Chou-Talalay combination index (CI) for the BTZ/VAS combination tested on LP1 and KMS-12-PE cells.

Cell Lines	VAS (μM)	BTZ (nM)	CI	Effects
LP1	2	2	1.382 ± 0.135	Antagonistic
	2	5	2.290 ± 0.788	Antagonistic
	2	10	1.289 ± 0.419	Antagonistic
	3	3	1.646 ± 0.063	Antagonistic
	4	4	1.516 ± 0.625	Antagonistic
KMS-12-PE	2	10	11.001 ± 5.607	Antagonistic
	4	5	1.074 ± 0.435	Additive
	4	10	1.007 ± 0.136	Additive

LP1 and KMS-12-PE cells were seeded in 96-well plates at a density of 5 × 10^4^ cells per well, then treated with the vehicle, or VAS (2 or 4 μM) alone, or BTZ alone (2 or 10 nM) or the combination of the two drugs. The viability was assessed by an MTS assay as described and the results analysed by the CompuSyn software to evaluate the combination index. The Chou-Talalay index offers a quantitative definition for additive effects (CI = 1.0), synergism (CI < 1.0), and antagonism (CI > 1.0) for drugs combination.

**Table 3:** Chou-Talalay combination index for AUR/BTZ combination tested on MM cells.

Cell Lines	AUR (μM)	BTZ (nM)	CI	Effects
OPM2	1	3.5	0.806 ± 0.216	Synergistic
	1	5	0.003 ± 0.001	Synergistic
	2	3.5	0.205 ± 0.067	Synergistic
H929	0.5	3.5	0.027 ± 0.002	Synergistic
L363	0.5	5	2.28 × 10^−6^ ± 5.00 × 10^−5^	Synergistic
	1	3.5	2.23 × 10^−5^ ± 1.29 × 10^−5^	Synergistic
	1	5	1.56 × 10^−6^ ± 3.89 × 10^−7^	Synergistic
	1	10	1.68 × 10^−9^ ± 1.95 × 10^−9^	Synergistic
JJN3	0.1	5	0.808 ± 0.082	Synergistic
	0.5	5	0.503. ± 0.109	Synergistic
LP1	2	5	0.068 ± 0.01	Synergistic
	3	5	0.488 ± 0.094	Synergistic
	1	10	0.047 ± 0.02	Synergistic
KMS-12-PE	1	5	1/∞	Synergistic
	1	10	3.48 × 10^−3^ ± 5.40 × 10^−4^	Synergistic
	2	5	1/∞	Synergistic
	2	10	1.60 × 10^−4^ ± 1.54 × 10^−4^	Synergistic

The indicated MM cells were seeded in 96-well plates at a density of 5 × 10^4^ cells per well, then treated with vehicle, AUR (0.1–3 μM) alone, BTZ alone (3.5–10 nM), or a combination of the two drugs at the indicated concentrations. The viability was assessed by an MTS assay as described and the results analysed by the CompuSyn software to evaluate the CI. The means ± SD calculated for each culture condition with triplicate samples are indicated.

**Table 4:** Statistical comparison between the treatments.

Cell Line	Culture Model	Treatment 1	Treatment 2	*p*-Value
H929	Suspension	AUR 2.5	AUR 5	<0.0001
		BTZ 20	BTZ 50	0.002
		BTZ 20	AUR 2.5 + BTZ 20	0.0002
		BTZ 20	AUR 5 + BTZ 20	0.0002
		AUR 2.5	AUR 2.5 + BTZ 20	0.2648
		AUR 5	AUR 5 + BTZ 20	0.0008
		AUR 2.5 + BTZ 20	AUR 5 + BTZ 20	0.0048
	Coculture	BTZ 20	BTZ 50	0.0006
		BTZ 20	AUR 2.5 + BTZ 20	<0.0001
		BTZ 20	AUR 5 + BTZ 20	<0.0001
		AUR 2.5	AUR 2.5 + BTZ 20	0.0034
		AUR 5	AUR 5 + BTZ 20	<0.0001
L363	Suspension	AUR 2.5	AUR 5	0.6863
		BTZ 20	BTZ 50	<0.0001
		BTZ 20	AUR 5 + BTZ 20	<0.0001
		AUR 5	AUR 5 + BTZ 20	0.1071
	Coculture	BTZ 20	BTZ 50	<0.0001
		BTZ 20	AUR 2.5 + BTZ 20	<0.0001
		AUR 5	AUR 5 + BTZ 20	0.0764

H929 and L363 BTZ-sensitive cells were seeded at a density of 10^5^ cells/well, then cultured in suspension or in coculture with HS-5 mesenchymal cells. Cells were treated 1 h later with AUR (μM), BTZ (nM) or a combo for 24 h at the indicated doses. Apoptosis induction was quantified as described in the legend of Figure 6a. The number of APO2.7-positive and CD10-negative cells (coculture) was recorded and used to calculate the *p*-values for the comparison between the treatments with the *t*-test.

**Table 5:** Statistical comparison between the culture models.

Cell Line	Treatment	Model 1	Model 2	*p*-Value
H929	AUR 2.5	Suspension	Coculture	<0.0001
	AUR 5	Suspension	Coculture	<0.0001
	BTZ 20	Suspension	Coculture	0.0033
	BTZ 50	Suspension	Coculture	<0.0001
	AUR 2.5 + BTZ 20	Suspension	Coculture	<0.0001
	AUR 5 + BTZ 20	Suspension	Coculture	<0.0001
L363	AUR 2.5	Suspension	Coculture	<0.0001
	AUR 5	Suspension	Coculture	<0.0001
	BTZ 20	Suspension	Coculture	<0.0001
	BTZ 50	Suspension	Coculture	0.0061
	AUR 5 + BTZ 20	Suspension	Coculture	<0.0001

H929 and L363 BTZ-sensitive cells were seeded at a density of 10^5^ cells/well, then cultured in suspension, or in coculture with HS-5 mesenchymal cells. Cells were treated 1 h later with AUR (μM), BTZ (nM) or in a combo for 24 h at the indicated doses. Apoptosis induction was quantified as described in the legend of Figure 6a. The number of APO2.7-positive and CD10-negative cells (coculture) was recorded and used to calculate the *p*-values for the comparison between the culture models with the *t*-test.

**Table 6:** Chou-Talalay combination index for AUR/BTZ combination tested on the HS-5 coculture and in spheroids.

Cell Line	Culture Model	AUR (μM)	BTZ (nM)	CI	Effects
H929	HS-5	2.5	20	0.139 ± 0.214	Synergistic
		5	20	0.061 ± 0.006	Synergistic
	3-D	5	50	0.728 ± 0.077	Synergistic
L363	HS-5	5	20	0.895 ± 0.028	Synergistic
	3-D	5	50	0.908 ± 0.036	Synergistic

H929 and L363 MM cells were seeded in 24-well plates at a density of 10^5^ cells per well on a HS-5 layer or cultured in spheroids for 6 days. The cells were then treated for 24 h with vehicle, AUR (2.5–25 μM), BTZ alone (20–100 nM), or the combination as indicated. The number of APO2.7-positive cells corresponding to apoptotic cells in the various culture conditions was recorded and used to calculate the Chou-Talalay index (CI) with the CompuSyn software. The indicated CI values are means ± SD from three independent experiments done with triplicate samples.

## Supplementary Materials

The following are available online at https://www.mdpi.com/2073-4409/9/11/2357/s1; Table S1: cell lines characteristics, origin, and authentication; Table S2: clinico-biological parameters of MM patients; Table S3: sequences of the primers used with RT-PCR for the analysis of NOX components and antioxidant enzymes expression in MM cell lines; Table S4: GEP analysis of NOX components and antioxidant enzymes from public datasets; Table S5: gene expression profiles of pro-oxidant enzymes in MM cell lines (ΔCt values); Table S6: gene expression profiles of antioxidant enzymes in MM cells (ΔCt values); Table S7: estimation of cleaved caspase 3 levels with immunoblotting; Table S8: resistance index for apoptosis induction in HS-5 coculture and 3-D vs. suspension; Figure S1: characteristics of MM cell lines; Figure S2: GEP expression analysis of antioxidant enzymes in molecular sub-groups of MM patients; Figure S3: Kaplan-Meyer curves of MM patients in relation with antioxidant enzymes expression; Figure S4: auranofin induces a caspase-dependent apoptosis and co-operates with bortezomib in L363 cells; Figure S5: western blot analysis of apoptotic and autophagic markers in LP1 and KMS-12-PE treated cells (original blots); Figure S6: the CAM-DR imposed by the culture on fibronectin-coated plates is reversed by the AUR/BTZ treatment; Figure S7: response of LP1 and KMS-12-PE cells cultured in suspension or on fibronectin-coated plates; Figure S8: morphological examination of MM cells cultured in spheroids.
