# Supplementary material for: ROS Overproduction Sensitises Myeloma Cells to Bortezomib-Induced Apoptosis and Alleviates Tumour Microenvironment-Mediated Cell Resistance
Source: Cells. 2020 Oct 26;9(11):2357. doi: 10.3390/cells9112357 (PMC7693395; doi:10.3390/cells9112357)
Supplement: Supplementary file 1 [file cells-09-02357-s001.pdf]

Supplementary informations

ROS overproduction sensitises myeloma cells to bortezomib-induced apoptosis and alleviates tumour microenvironment-mediated cell resistance

Mélody Caillot, Florence Zylbersztejn, Elsa Maitre, Jérôme Bourgeais, Olivier Hérault and Brigitte Sola

1. Supplementary materials and methods

1.1. Antibodies

Antibodies (Abs) against NOX2 (or gp9^{phox}, ab80508), RAC1 (ab1555938) were purchased from abcam (Cambridge, UK). The Ab against β-actin (sc-4778) was obtained from Santa Cruz Biotechnology (Santa Cruz, CA, USA). Peroxidase-conjugated secondary Abs were purchased from Pierce Biotechnology (ThermoFisher Scientific, Waltham, MA, USA).

1.2. LP1-derived cell lines

Green fluorescent protein (GFP) and cyclin D1-GFP expressing cells have been described previously [1]. Briefly, LP1 MM cells were transfected by electroporation with expressing plasmids coding for GFP or a fusion cyclin D1-GFP protein. Transfected clones were obtained by limiting dilution and maintained under selective pressure with G418 (500 ng/ml, Lonza). GFP expression was checked regularly by flow cytometry.

1.3. Human samples microarray analysis

Microarray expression profiles were obtained from the Gene Expression Omnibus (GEO) database (ncbi.nlm.nih.gov/geo/) using the accession number GSE24080, which contained 559 samples of patients with MM (MAQC Consortium, 2010). To generate the gene expression profiles (GEP), the expression matrix and microarray platform annotation files were downloaded. For statistical analyses, log-rank test was performed using the Cutoff Finder software (http://molpath.charite.de/cutoff) [2].

2. Supplementary tables

Table S1. Cell lines, characteristics, origin and authentication

**Cell lines**	**Molecular subgroup**	***TP53* status**	**Origin**	**Reference**
JJN3	MF	abn	DSMZ* (ACC-541)	[3]
KMS-12-PE	CD1/2	abn	C. Pellat	[4]
LP1	MS	abn	R. Bataille	[3]
L363	MF	abn	C. Pellat	[5]
MM.1S	MF	wt	DSMZ (ACC-41)	[6]
H929	MS	wt	R. Bataille	[3]
OPM2	MS	abn	D. Bouscary	[7]
8226	MF	abn	DSMZ (ACC-402)	[3]
U266	CD1/2	abn	R. Bataille	[3]

* Cell lines were either generous gifts of collaborators or were purchased from DSMZ (Leibniz Institute, Braunschweig, Germany). Cell authentication was based on short tandem repeat (STR) profiling by DSMZ. Abbreviations: abn, abnormal; wt, wild-type. The *TP53* status of MM cell lines was based on [8].

Table S2. Clinico-biological parameters of MM patients

**Patient (#)**	**Age (y)**	**Gender**	**Plasma cells (%)**	**IgH/L**	**CMF***	**ISS**	**CRAB symptoms**	**Chromosome abnormalities**
1	58	F	22	IgG/λ	CD28+<br>CD20+<br>CD56-	stade 3	yes	t(14;20)
2	87	F	37	IgG/κ	CD28-<br>CD20-<br>CD56+	nd	yes	t(4;14)<br>gain 1q31
3	52	F	23	IgG/κ	CD28+<br>CD20-<br>CD56-	stade 2	yes	t(4;14)<br>gain 1q
4	74	F	25	IgG/κ	CD28-<br>CD20-<br>CD56-	stade 2	yes	nd

Analysed by flow cytometry, tumour cells were all found CD38+, CD138+, CD117-, CD19-. Only the positivity/negativity of CD28, CD20 and CD56 that may change are indicated. Abreviations: CRAB, for the most common symptoms of myeloma (hypercalcemia, renal failure, anemia and bone lesions); ISS, International Staging System; nd, not done.

Table S3. Sequences of the primers used with RT-PCR for the analysis of NOX components and antioxidant enzymes expression in MM cell lines

**Gene**	**Probe**	**Forward primer* 5'-3'**	**Reverse primer 5'-3'**
ACTB	gctggaag	attggcaatgagcggttc	cgtggatgccacaggact
CAT	ctccagca	cgcagttcggttctccac	gggtcccgaactgtgtca
CYBA (p22^{phox})	gcagtgga	gagcggcatctacctactgg	tgatggtgcctccgatct
CYBB (NOX2)	tggcagag	gaagaaaggcaaacacaacaca	ccccagccaaaccagaat
DUOX1	ctggaga	cacctcctggagaccttttc	gtcggcctggttgatgtc
DUOX2	cttcccca	tgcatattccccaacgtctt	ggtctggaagaaccaccaatag
GAPDH	cttcccca	agccacatcgctcagacac	gcccaatacgaccaaatcc
GLRX var1&2	cagccacc	ggcttctggaatttgtcgat	tgcatccgcctatacaatctt
GLRX2 var1	ctccatcc	gtggcactcgctggaatc	cgtcgctaaattctccaaagat
GLRX2 var2	ggcggcgg	gctggtttggagcaggag	ccaaagatgatgatgtattgctct
GLRX3	tggtgga	tcctcaagaaccacgctgt	tgagaagatatcaaaactgctaaactg
GLRX5	ctccagca	gtgataactggggcgttgtt	actcaggcatgcacagca
GPX1var1	ccaccacc	caaccagtttgggcatcag	gttcacctcgcacttctcg
GPX1var2	ctcctcct	cccttgtttgtggttagaacg	gagagaagggcagctagaacc
GPX2	caggagaa	gtccttggcttcccttgc	tgttcaggatctcctcattctg
GPX3	aggtggag	cagagatccttcctaccctcaa	ccctttctcaaagagctgga
GPX4 var1/2/3	ctgcccca	tacggacccatggaggag	ccacacacttgtggagctagaa
GPX7	ctccttcc	ccatcctgccttcaagtacc	ttccatctggggctactagg
GSR	gctggaag	tgccagcttaggaataaccag	cctgcaccaacaatgacg
NCF1 (p47^{phox})	ccagccag	cctgctgggctttgagaa	gacaggtcctgccatttcac
NCF2 var1/2 (p67^{phox})	caggcagc	ctctgggtttgcccctct	tctctggggttttcggtct
NCF4 var1/2 (p40^{phox})	ccaggca	tttgcagagcaagctggag	tcctgtttcacacccacgta
NOX1	ctgctggg	aaggatcctccggttttacc	tttggatgggtgcataacaa
NOX3	cttcccca	cgagagctacctcaaccctgt	tgacgcctgctattgtcctt
NOX4	ggctgctg	gctgacgttgcatgtttcag	cgggagggtgggtatctaa
NOX5	gcagccag	cccttcaccatcagcagtg	tgtttgtccactggccttg
NOXA1	cagcaggt	gtcacggcttggtcaaatg	gccaggctgtgcttcaac
NOXO1	cagccacc	caggagagcctggacgtg	ctgccggtcttcgttctc
PRDX1 var1/2/3	ccagccag	cactgacaaacatggggaagt	tttgctcttttggacatcagg
PRDX2 var1	cttcccca	gccttccagtacacagacgag	gttgggcttaatcgtgtcact
PRDX2 var3	cagcctcc	gcaactcagatgcaactctatctact	tgaactggagtttccatcttcat
PRDX3 var1/2	ctgcttcc	ctggacaccggattctccta	gggtgatctactgatttaccttctg
PRDX4	actgggaa	gcacctaagcaaagcgaaga	aaattctccatcgatcacagc
PRDX5 var1/3	ctccttcc	tcctggctgatcccactg	atgccatcctgtaccaccat
PRDX5 var2	ctccttcc	cacccctggatgttccaa	ggacaccagcgaatcatctagt
PRDX6	gctccagg	caatagacagtgttgaggaccatc	tttctgtgggctcttcacaa
RPL13A	ccagccgc	caagcggatgaacaccaac	tgtggggcagcatacctc
SOD1	cttcccca	gcatcatcaatttcgagcag	caggccttcagtcagtcctt
SOD2	ctgctggg	tccactgcaaggaacaacag	taagcgtgctcccacacat

SOD3	aggagctg	ctctcttttcaggagagaaagctc	aacacagtagcgccagcat
TXN	ggctgctg	ttacagccgctcgtcaga	ggcttcctgaaaagcagtctt
TXNRD1	cttcctgc	tcaccccagttgcaatcc	ggttggaacattttcatagtcaca

* PCR primers and UPL probes were designed using ProbeFinder software (Roche Applied software, Penzberg, Germany).

Table S4. GEP analysis of NOX components and antioxidant enzymes from public datasets

Gene (symbol)	Protein (name)	Probe
NOX1	NADPH oxidase 1	206418_at
CYBB	Cytochrome b-245 or NOX2	203922_s_at
NOX3	NADPH oxidase 3	221089_at
NOX4	NADPH oxidase 4	219773_at
NOX5	NADPH oxidase 5	1553023_a_at
DUOX1	Dual oxidase 1	1553023_a_at
DUOX2	Dual oxidase 2	219727_at
NCF1	Neutrophil cytosolic factor 1 or NOXO2 or $p47^{phox}$	M55067_at*
NCF2	Neutrophil cytosolic factor 2 or NOXA2 or $p67^{phox}$	209949_at
NCF4	Neutrophil cytosolic factor 4 or $p40^{phox}$	205147_x_at
CYBA	Cytochrome b-245 alpha chain or $p22^{phox}$	203028_s_at
NOXA1	NADPH oxidase activator 1	232373_at
NOXO1	NADPH oxidase organizer 1	235329_at
CAT	Catalase	201432_at
GLRX	Glutaredoxin	206662_at
GLRX2	Glutaredoxin 2	219933_at
GLRX3	Glutaredoxin 3	209080_x_at
GLRX5	Glutaredoxin 5	221932_s_at
GPX1	Glutathione peroxidase 1	200736_s_at
GPX2	Glutathione peroxidase 2	202831_at
GPX3	Glutathione peroxidase 3	201348_at
GPX4	Glutathione peroxidase 4	201106_at
GPX5	Glutathione peroxidase 5	208028_s_at
GPX7	Glutathione peroxidase 7	213170_at
GSR	Glutathione-disulfide reductase	225609_at
PRDX1	Peroxiredoxin 1	208680_at
PRDX2	Peroxiredoxin 2	39729_at
PRDX3	Peroxiredoxin 3	201619_at
PRDX4	Peroxiredoxin 4	201923_at
PRDX5	Peroxiredoxin 5	1560587_s_at
SOD1	Superoxide dismutase 1	200642_at
SOD2	Superoxide dismutase 2	1566342_at
SOD3	Superoxide dismutase 3	205236_x_at
TXN	Thioredoxin	208864_s_at
TXN2	Thioredoxin 2	209077_at
TXNRD1	Thioredoxin reductase 1	201266_at

* For this probe, we used Tarte's datasets [9], for the others, Zhan's datasets [10]. *GPX2*, *GPX3*, *GPX5*, *TXNRD2*, *SOD3* are not expressed in normal nor tumour plasma cells.

Table S5. Gene expression profiles of pro-oxidant enzymes in MM cell lines (ΔCt values)

Gene/Cell	JJN3	KMS-12-PE	LP1	L363	MM.1S	H929	OPM2	8226	U266
CYBB	15.79	12.33	17.54	15.87	10.69	7.26	13.54	5.58	16.63
NOX4	-	-	-	12.77	-	12.63	16.36	15.59	17.74
NOX5	-	-	-	-	11.69	16.22	-	16.47	-
DUOX2	-	-	15.62	16.83	-	16.18	-	-	-
CYBA	4.92	5.40	5.61	3.91	3.94	3.69	3.61	3.54	5.10
NCF1	14.83	6.69	13.45	14.42	5.60	3.68	16.84	7.01	12.37
NCF2	6.25	12.38	-	13.15	6.18	8.22	16.75	5.39	9.97
NCF4	-	-	-	-	16.09	15.17	-	13.15	-
NOXA1	12.85	12.59	13.76	15.96	-	13.56	17.47	13.05	-
NOXO1	14.89	15.62	13.94	13.65	13.32	15.31	14.07	12.83	14.21

Gene expression data for each gene and each cell line were normalised to internal control genes (*GAPDH*/*RPL13A*/*ACTB*). *NOX1* is expressed only in L363 and U266 cells (ΔCt = 17.58 and 17.82, respectively), *NOX3* is not expressed, DUOX1 is expressed in MM.1S and U266 (ΔCt = 14.11 and 14.21, respectively). -, not expressed.

Table S6. Gene expression profiles of antioxidant enzymes in MM cell lines (ΔCt values)

Gene/Cell	JJN3	KMS-12-PE	LP1	L363	MM.1S	H929	OPM2	8226	U266
SOD1	2.07	1.75	2.49	3.00	1.77	2.05	2.34	1.25	0.76
SOD2	5.24	4.60	6.64	6.19	6.15	5.33	4.99	5.14	4.92
CAT	11.13	9.48	11.02	11.92	11.02	10.01	10.55	10.25	11.00
TXN	5.06	2.74	5.26	4.09	4.33	3.86	4.34	3.89	3.92
TXNRD1	7.49	7.23	7.11	7.21	7.44	6.75	7.28	6.84	6.98
GLRX1 var1/2	8.47	6.91	9.08	7.15	6.42	6.99	9.71	6.65	6.27
GLRX2 var1	15.66	15.67	13.39	14.70	15.47	14.64	13.40	13.94	13.87
GLRX2 var2	7.33	7.65	5.90	6.18	6.79	6.97	6.87	6.15	7.26
GLRX3	7.25	7.00	7.33	6.70	6.84	7.17	7.34	8.17	6.54
GLRX5	-	12.68	13.19	13.55	13.68	11.70	12.30	12.26	12.69
GPX1 var1	7.87	-	8.04	6.61	8.39	6.48	-	6.45	7.00
GPX1 var2	10.12	11.42	10.61	10.81	11.73	9.72	12.07	10.43	10.82
GPX4 var1/2/3	5.82	3.83	5.60	5.30	4.89	4.95	6.30	5.87	3.77
GPX7	5.43	2.46	9.41	13.31	7.04	5.39	13.95	2.36	12.69
GSR	5.40	2.77	4.10	3.81	3.70	4.32	3.70	4.11	4.68
PRDX1 var1/2/3	2.63	2.71	6.47	3.24	1.90	2.13	3.16	1.06	1.60
PRDX2 var1	3.64	2.98	3.65	2.88	3.96	3.15	4.06	3.62	4.49
PRDX2 var3	12.51	11.03	12.28	13.03	12.96	12.33	12.72	12.85	13.43
PRDX3 var1/3	4.00	3.46	2.70	1.95	3.47	2.79	3.35	3.39	3.04
PRDX4	3.85	4.21	3.80	3.46	4.09	3.79	3.89	2.42	4.99
PRDX5 var1/3	2.85	1.93	2.52	2.19	2.59	2.01	2.53	1.30	2.24
PRDX5 var2	7.65	7.31	7.02	6.93	6.12	7.14	6.97	5.65	7.01
PRDX6	12.47	10.58	11.37	12.58	13.19	11.14	10.83	10.91	12.51

Gene expression data for each gene and each cell line were normalised to internal control genes (*GAPDH*/*RPL13A*/*ACTB*). *SOD3* is not expressed, *GPX2* is expressed only in MM.1S cells (ΔCt = 16.62), *GPX3* is expressed only in JJN3 (ΔCt = 15.63). -, not expressed.

Table S7. Estimation of cleaved caspase 3 levels with immunoblotting

**KMS-12-PE**		V	A1	A2	A3	B2.5	B5	B10	A/B
Cl. caspase 3	17 kDa	0.00	1.10	nd	nd	0.00	0.00	1.00	1.64
**LP1**		V	A1	A2	A3	B2.5	B5	B10	A/B
Cl. caspase 3	19 kDa	0.00	1.00	nd	nd	0.00	0.00	4.21	2.25
	17 kDa	0.00	1.00	nd	nd	0.00	0.00	4.75	2.64

KMS-12-PE and LP1 cells were treated with vehicle (V), 1-3 µM AUR (A1-A3), 2.5-10 nM BTZ (B2.5-B10), or 1 µM AUR plus 2.5 nM BTZ (A/B) for 24 h. Whole-cell protein extracts were prepared and separated by SDS-PAGE. Proteins were blotted and analysed with an anti-cleaved caspase (#9664 from Cell Signaling Technologies). The anti-cleaved caspase 3 Ab detects two forms of 19 and 17 kDa. An anti-β-actin Ab was used as a control of loading and transfer. The levels of each protein were estimated by densitometry and normalised against the β-actin level. The calculated ratios were collected in the table. Abbreviations: nd, not done; Cl., cleaved.

Table S8. Resistance index for apoptosis induction in HS-5 coculture and 3-D culture vs. suspension

**Cell line**	**Culture model**	**Treatment**	**Resistance index**	***p*-value**
H929	HS-5 cells	AUR	2.49 ± 0.04	< 0.0001
		BTZ	1.62 ± 0.13	0.0022
	3-D	AUR	16.67 ± 0.27	< 0.0001
		BTZ	2.43 ± 0.06	< 0.0001
L363	HS-5 cells	AUR	1.79 ± 0.05	< 0.0001
		BTZ	1.19 ± 0.01	0.0012
	3-D	AUR	15.86 ± 0.39	< 0.0001
		BTZ	1.26 ± 0.02	< 0.0001

H929 and L363 MM cells were cultured either in suspension, or in co-culture with HS-5 mesenchymal cells, or in spheroids as described in the main text. Cells were treated with vehicle as a control, 5 µM auranofin (AUR) or 50 nM bortezomib (BTZ) for 24 h. Cells were then incubated with an anti-APO2.7-PE-conjugated Ab and an anti-CD10-APC conjugated Ab and analysed by image cytometry. APO2.7-positive/CD10-negative cells corresponding to apoptotic MM cells were recorded. The experiments were performed three times. The index of resistance was calculated as the number of apoptotic cells in the various culture models relative to the culture in suspension. The means ± SD are indicated in the table. The *p*-values were calculated with the *t*-test.

Supplementary figures

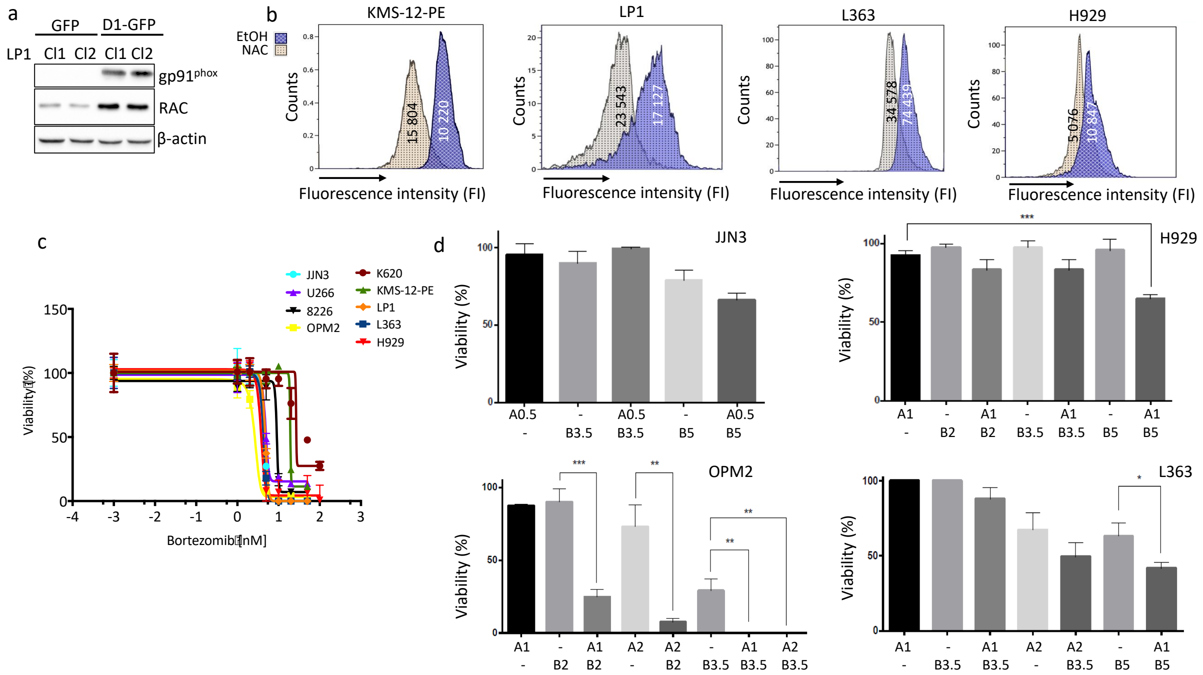

Figure S1.

(**a**) LP1 parental cells were stably transfected with expression plasmids and selected with G418. They synthesised either GFP or a fusion cyclin D1-GFP protein as described previously [1]. Two clones (Cl1, Cl2) were studied for each series. Whole-cell protein extracts were obtained from cultured cells and separated by SDS-PAGE. Proteins were blotted and analysed with the indicated Abs. Anti-β-actin Ab was used as a control of loading and transfer; (**b**) Cultured KMS-12-PE, LP1, L363 and H929 cells were analysed after a 1 mM NAC-treatment (or 0.01% EtOH as a vehicle) overnight for the determination of basal production of ROS with the NucleoCounter NC-3000 image cytometer after CellROX Deep Red staining as described in Figure 3c. At least, 10^4 cells were analysed for each culture condition. The experiment has been repeated twice with similar results. The means of fluorescence intensity (MFI) from one representative experiment are indicated on the graph; (**c**) The panel of MM cell lines used in this study was treated with various concentrations of BTZ (0.5-50 nM) or vehicle (0.01% DMSO) for 48 h and cell viability assayed using an MTS assay (CellTiter 96®AQ_{ueous} One Solution Cell Proliferation Assay, Promega). The absorbance (OD at 490 nM) of each cell line treated with the drug is expressed relative to that of the corresponding cell treated with vehicle (defined as 100%). For each set of culture conditions, the means ± SD of triplicate ratios are indicated on the graph. The curves were drawn with the Prism v6.0 software; (**d**) JJN3, H929, OPM2 and L363 cell lines were treated with auranofin alone (A, 0.5-2 µM), BTZ alone (B, 2-5 nM) or the combination and their viability assessed as before with the MTS assay. Bar graphs correspond to the means ± SD of triplicate samples. The experiment has been performed three times. * $p < 0.05$, ** $p < 0.01$, and *** $p < 0.001$ in the *t*-test.

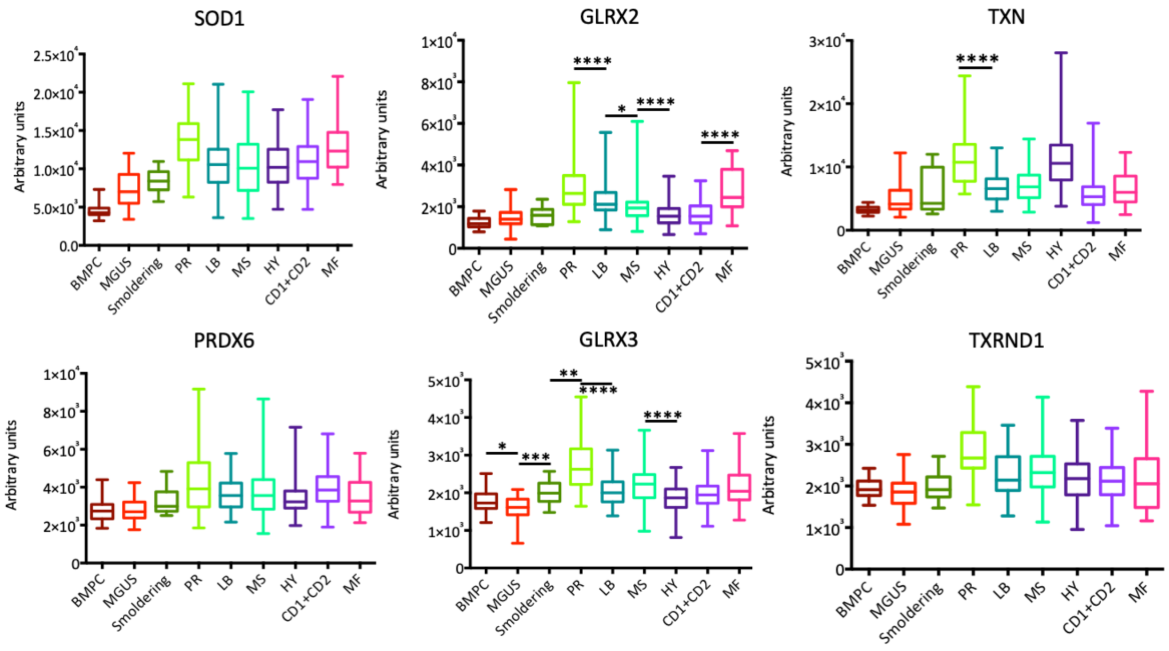

Figure S2. GEP expression analysis of antioxidant enzymes in molecular subgroups of MM patients. We used the Amazonia! tool (http://*amazonia.transcriptome.eu/*) for GEP analysis of antioxidant enzymes from Zhan datasets [10, Table S3]. *SOD1*, *GLRX2, TXN, PRDX6, GLRX3, TXRND1* genes are overexpressed in MM cells compared to BMPC, MGUS and smouldering myeloma (Figure 2). The boxplots show the expression signal of the indicated probes in arbitrary units for BMPC (n = 22), MGUS patients (n = 44), patients with smouldering MM (n = 12), or overt MM (n = 414). MM patients were classified at diagnosis according to their molecular subgroups: PR (n = 47), LB (n = 58), MS (n = 68), HY (n = 116), CD1/2 (n = 88), MF (n = 37). * $p < 0.05$, ** $p < 0.01$, *** $p < 0.001$, **** $p < 0.0001$ with the Mann-Whitney test calculated with the Prism software.

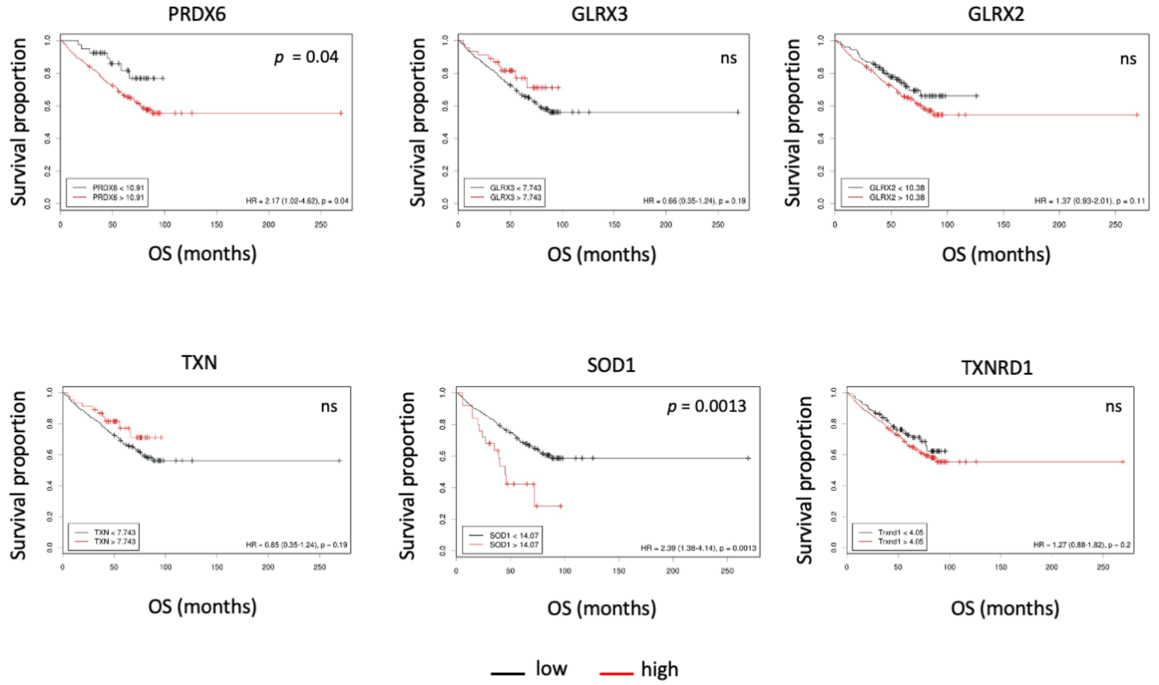

Figure S3. Kaplan-Meyer curves of patients in relation with antioxidant enzymes expression. The GenBank dataset study (GEO: GSE24080; n=559) analysing overall survival of patients was retrospectively analysed for *PRDX6*, *GLRX2/3*, *SOD1*, *TXN*, and *TRXND1* expression. The GEO2R software required analysis options called Benjamini & Hochberg. Log-rank analysis comparing different groups of patients (separated in high and low

expression) according to the relative gene expression (low gene expression (black line); high gene expression (red line). For statistical analyses, log-rank test was performed using the Cutoff Finder software (http://molpath.charite.de/cutoff) [2]. Significant *p*-values are indicated on the graph; ns, not significant.

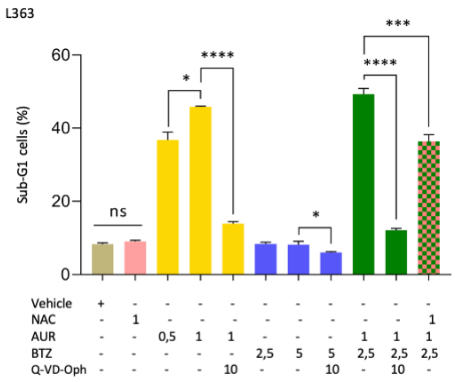

Figure S4. Auranofin induces a caspase-dependent apoptosis and co-operates with bortezomib in L363 cells. L363 BTZ-sensitive cells were treated for 24 h with vehicle (V, 0.01% DMSO), AUR (0.5-1 µM), BTZ (2.5-5 nM) or AUR/BTZ combination. In some cases, cells were treated with 10 µM Q-VD-OPh for 1 h or 1 mM NAC for 12 h before AUR/BTZ treatments. Treated-cells were fixed in EtOH and incubated in a buffer containing RNase A and DAPI. Cell cycle was analysed by image cytometry (NucleoCounter NC-3000, ChemoMetec). At least 10^4 cells were analysed for each culture condition. The number of cells in each phase of the cell cycle (sub-G1, GO/G1, S, G2/M) was determined by the Kaluza software (Beckman Coulter). Data were exported and analysed with the PRISM software. Histograms representing the means ± SD of the percentage of apoptotic cells were drawn with PRISM and the *p*-values calculated by the same software with the *t*-test. The experiment has been performed three times with triplicate samples for each cell line, a representative experiment is shown. ns, not significant; *, $p < 0.05$; ***, $p < 0.001$; ****, $p < 0.0001$ with the *t*-test.

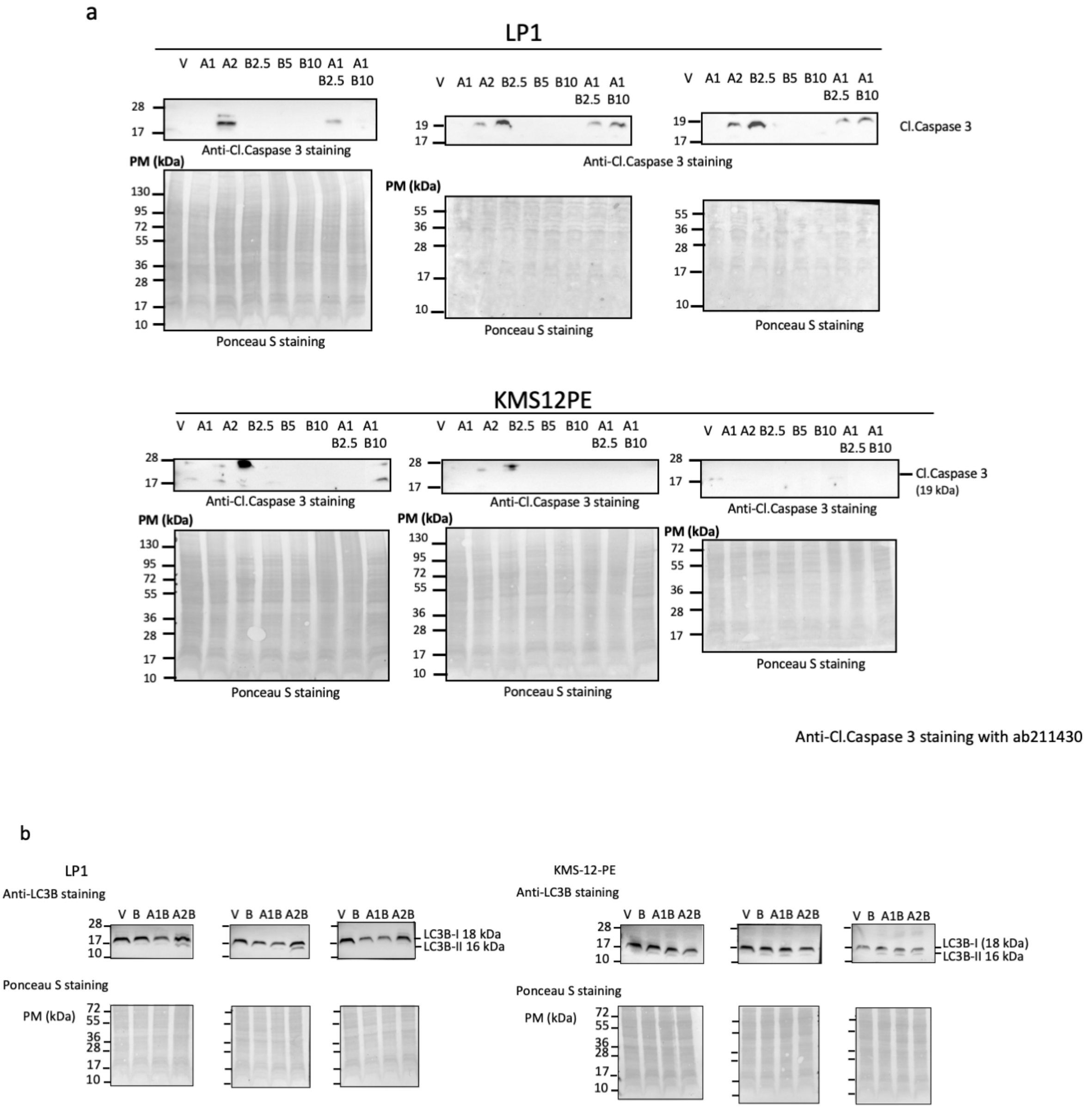

Figure S5. Original blots presented in Figure 5a,b. Whole-cell extracts were prepared from the indicated cell lines treated with the indicated drugs. Proteins were subjected to SDS-PAGE, transferred onto nitrocellulose sheets that were stained with Ponceau S. The blots were then cut into strips that were incubated with the indicated Abs.

a

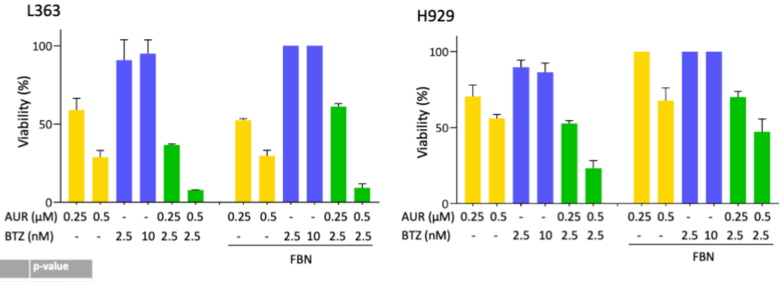

b

Statistical comparison between the treatments

Cell Line	Culture model	Treatment 1	Treatment 2	p-value
L363	Suspension	AUR 0.25	AUR 0.5	0.0380
	Suspension	BTZ 2.5	BTZ 10	0.9658
	Suspension	AUR 0.25	AUR 0.25 + BTZ 2.5	0.0110
	Suspension	AUR 0.5	AUR 0.5 + BTZ 2.5	0.0189
	Suspension	BTZ 2.5	AUR 0.25 + BTZ 2.5<br>AUR 0.5 + BTZ 2.5	0.0005
	Suspension	AUR 0.25 + BTZ 2.5	AUR 0.5 + BTZ 2.5	<0.0001
	FBN	AUR 0.25	AUR 0.5	0.0034
	FBN	BTZ 2.5	BTZ 10	0.3739
	FBN	AUR 0.25	AUR 0.25 + BTZ 2.5	0.0112
	FBN	AUR 0.5	AUR 0.5 + BTZ 2.5	0.0012
	FBN	BTZ 2.5	AUR 0.25 + BTZ 2.5<br>AUR 0.5 + BTZ 2.5	<0.0001
	FBN	AUR 0.25 + BTZ 2.5	AUR 0.5 + BTZ 2.5	<0.0001
H929	Suspension	AUR 0.25	AUR 0.5	0.0344
	Suspension	BTZ 2.5	BTZ 10	0.4867
	Suspension	AUR 0.25	AUR 0.25 + BTZ 2.5	0.0166
	Suspension	AUR 0.5	AUR 0.5 + BTZ 2.5	0.0006
	Suspension	BTZ 2.5	AUR 0.25 + BTZ 2.5<br>AUR 0.5 + BTZ 2.5	<0.0001
	Suspension	AUR 0.25 + BTZ 2.5	AUR 0.5 + BTZ 2.5	0.0007
	FBN	AUR 0.25	AUR 0.5	0.0027
	FBN	BTZ 2.5	BTZ 10	0.4411
	FBN	AUR 0.25	AUR 0.25 + BTZ 2.5	0.0002
	FBN	AUR 0.5	AUR 0.5 + BTZ 2.5	0.0412
	FBN	BTZ 2.5	AUR 0.25 + BTZ 2.5<br>AUR 0.5 + BTZ 2.5	<0.0001
	FBN	AUR 0.25 + BTZ 2.5	AUR 0.5 + BTZ 2.5	0.0124

Statistical comparison between the culture models

Cell Line	Treatment	Model 1	Model 2	p-value
L363	AUR 0.25	Suspension	FBN	0.3428
	AUR 0.5	Suspension	FBN	0.8266
	BTZ 2.5	Suspension	FBN	0.2722
	BTZ 10	Suspension	FBN	0.3739
	AUR 0.25 + BTZ 2.5	Suspension	FBN	<0.0001
	AUR 0.5 + BTZ 2.5	Suspension	FBN	0.5195
H929	AUR 0.25	Suspension	FBN	0.0024
	AUR 0.5	Suspension	FBN	0.0851
	BTZ 2.5	Suspension	FBN	0.0190
	BTZ 10	Suspension	FBN	0.0173
	AUR 0.25 + BTZ 2.5	Suspension	FBN	0.019
	AUR 0.5 + BTZ 2.5	Suspension	FBN	0.0132

c

Cell Line	Treatment	Resistance index
L363	AUR 0.25 µM	1.10 ± 0.21
	AUR 0.5 µM	1.05 ± 0.009
	BTZ 2.5 nM	1.14 ± 0.12
	BTZ 10 nM	1.06 ± 0.10
	AUR 0.25 µM + BTZ 2.5 nM	1.67 ± 0.03
	AUR 0.5 µM + BTZ 2.5 nM	1.25 ± 0.24
H929	AUR 0.25	1.43 ± 0.15
	AUR 0.5	1.21 ± 0.10
	BTZ 2.5	1.12 ± 0.06
	BTZ 10	1.16 ± 0.08
	AUR 0.25 + BTZ 2.5	1.33 ± 0.02
	AUR 0.5 + BTZ 2.5	2.04 ± 0.20

Figure S6. The CAM-DR imposed by the culture on fibronectin-coated culture plates is reversed by the AUR/BTZ combined treatment. (**a**) H929 and L363 cells sensitive to BTZ were assayed for response to AUR alone (0.25-0.5 µM), BTZ (2.5-10 nM) and AUR/BTZ combination with an MTS assay. Bar graphs correspond to the means + SD of triplicate ratios. The experiment has been performed three times, a representative one is shown. (**b**) The *t*-test was used for comparing two groups of values, the one-way ANOVA for comparing more than two groups. The *p*-values are indicated in the tables; (**c**) The resistance index was calculated for each treatment as the percentage of viability of treated cells cultured on FBN-coated plates relative to that of cells cultured in suspension.

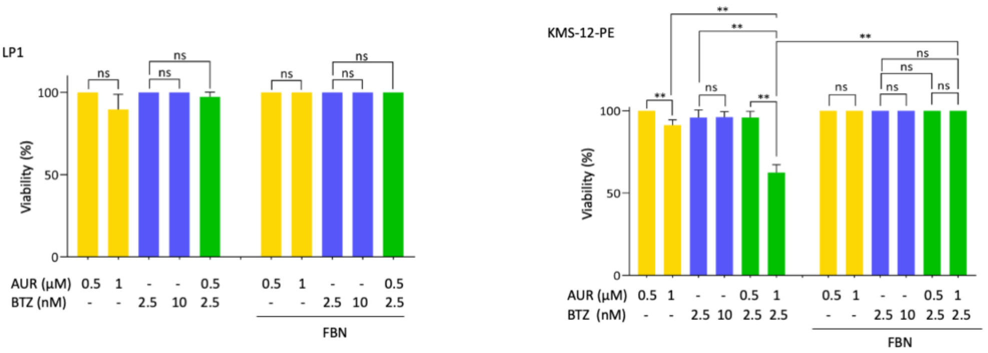

Figure S7. Response of LP1 and KMS-12-PE cells cultured in suspension or on fibronectin-coated plates. Both cell lines less cultured in suspension or on fibronectin (FBN)-coated culture plates sensitive to BTZ were assayed for response to AUR alone (0.5-1 µM), BTZ (2.5-10 nM) and AUR/BTZ combination with an MTS assay. Bar graphs correspond to the means + SD of triplicate ratios. The experiment has been performed three times, a representative one is shown. ns, not significant; **, $p < 0.01$ with the *t*-test

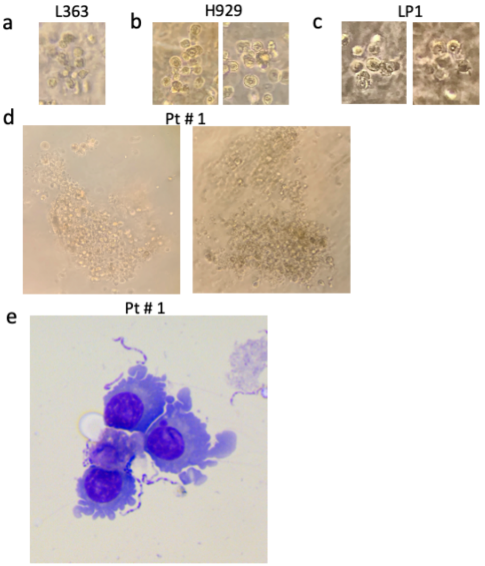

Figure S8. Morphological examination of MM cells cultured in spheroids. L363 (**a**), H929 (**b**) and LP1 (**c**) cells were cultured in a 3-D reconstructed bone marrow-based culture model as described by Huang *et al*. [11]. The morphology of spheroids was examined by phase-contrast microscopy six days after seeding. Images were taken with a x 320 magnification. (**d**) Mononuclear cells of MM patient #1 were directly culture in the 3-D model recapitulating the bone marrow niche. They formed spheroids as soon as two days before seeding. The images were obtained after a six-day culture period by phase-contrast microscopy examination with a x 100 magnification. (**e**) The presence of plasma cells in spheroids from Pt #1 was checked by May-Grünwald/Giemsa staining and microscopy examination with a x 1,000 magnification.

Supplementary references

1. Bustany, S.; Cahu, J.; Guardiola, P.; Sola, B. Cyclin D1 sensitizes myeloma cells to endoplasmic reticulum stress-mediated apoptosis by activating the unfolded protein response pathway. *BMC Cancer* **2015**, *15*, 262.
2. Budczies, J.; Klauschen, F.; Sinn, B.V.; Györffy, B.; Schmitt, W.D.; Darb-Esfahani, S.; Denkert, C. Cutoff Finder: A comprehensive and straightforward web application enabling rapid biomarker cutoff optimization. *PLoS One* **2012**, 12, e51862.
3. Drexler, H.G.; Matsuo, Y. Malignant hematopoietic cell lines: in vitro models for the study of multiple myeloma and plasma cell leukemia. *Leuk. Res*. **2000**, *24*, 681-703 .
4. Namba, M.; Ohtsuki, T.; Mori, M.; Togawa, A.; Wada, H.; Sugihara, T.; Tawata, Y.; Kimoto, T.l. Establishment of five human myeloma cell lines. *In Vitro Cell. Dev. Biol*. **1989**, *25*, 723-729.
5. Diehl, V.; Schaadt, M.; Kirchner, H.; Hellriegel, K.P.; Gudat, F.; Fonatsch, C.; Laskewitz, E.; Guggenheim, R. Long-term cultivation of plasma cell leukemia cells and autologous lymphoblasts (LCL) in vitro: a comparative study. *Blut* **1978**, *36*, 331-338.
6. Goldman-Leikin, R.E.; Salwen, H. R.; Herst, C. V.; Variakojis, D.; Bian, M. L.; Le Beau, M. M.; Selvanayagan, P.; Marder, R.; Anderson, R.; Weitzman, S. Characterization of a novel myeloma cell line, MM.1. *J. Lab. Clin. Med.* **1989**, *113*, 335-345.
7. Katagiri, S.; Yonezawa, T.; Kuyama, J.; Kanayama, Y.; Nishida, K.; Abe, T.; Tamaki, T.; Ohnishi, M.; Tarui, S. Two distinct human myeloma cell lines originating from one patient with myeloma. *Int. J. Cancer* **1985**, *36*, 241-246.
8. Moreaux, J.; Klein, B.; Bataille, R.; Descamps, G.; Maïga, S.; Hose, D.; Goldschmidt, H.; Jauch, A.; Rème, T.; Jourdan, M.; Amiot, M.; Pellat-Deceunynck, C. A high-risk signature for patients with multiple myeloma established from the molecular classification of human myeloma cell lines. *Haematologica* **2011**, 96, 574-582.
9. Tarte, K.; Zhan, F.; De Vos, J.; Klein, B.; Shaughnessy, J.Jr. Gene expression profiling of plasma cells and plasmablasts: toward a better understanding of the late stages of B-cell differentiation. *Blood* **2003**, *102*, 592-600.
10. Zhan, F.; Huang, Y.; Colla, S.; Stewart, J.P.; Hanamura, I.; Gupta ,S.; Epstein, J.; Yaccoby, S.; Sawyer, J.; Burington, B.; Anaissie, E.; Hollmig, K.; Pineda-Roman, M.; Tricot, G.; van

Rhee, F.; Walker, R.; Zangari, M.; Crowley, J.; Barlogie, B.; Shaughnessy, J.D.Jr.. The molecular classification of multiple myeloma. *Blood* **2006**, 108, 2020-2028.

11. Huang, Y.H.; Molavi, O.; Alshareef, A.; Haque, M.; Wang, Q.; Chu, M.P.; Venner, C.P.; Sandhu, I.; Peters, A.C.; Lavasanifar, A.; Lai, R. Constitutive activation of STAT3 in myeloma cells cultured in a three-dimensional, reconstrucyed bone marrow model. *Cancers* **2018**, 10, 206.
